# Discovery of novel melatonin–mydroxyquinoline hybrids as multitarget strategies for Alzheimer’s disease therapy

**DOI:** 10.3389/fchem.2024.1374930

**Published:** 2024-04-16

**Authors:** Wei Wang, Tingting Pan, Rui Su, Mingbin Chen, Wandi Xiong, Congjun Xu, Ling Huang

**Affiliations:** ^1^ Key Laboratory of Tropical Biological Resources of Ministry of Education, School of Pharmaceutical Sciences, Hainan University, Haikou, China; ^2^ School of Pharmaceutical Sciences, Sun Yat-Sen University, Guangzhou, China

**Keywords:** melatonin, Alzheimer’s disease, natural products, multitarget strategies, hydrids

## Abstract

Alzheimer’s disease (AD) is a neurodegenerative disease that seriously affects human health, and current treatment strategies are far from meeting clinical needs. Inspired by multi-target drug design strategies, a series of novel natural products-based melatonin–hydroxyquinoline hybrids were designed and synthesized, targeting anti-oxidation and metal-chelating at the same time. Most of the compounds showed significant oxygen radical absorbance capacity and Aβ_1–42_ aggregation inhibition. Moreover, the compounds possess good blood-brain barrier permeability. **6b** and **6c** have a good ability to alleviate oxidative stress induced by hydrogen peroxide. **6b** and **6c** possess metal-chelating properties with the chelation ratio being 2:1. Furthermore, **6b** can significantly mitigate metal-induced Aβ aggregation. This work may provide a new multi-target treatment strategy for Alzheimer’s disease.

## 1 Introduction

Alzheimer’s disease (AD) is a neurodegenerative disease characterized by memory loss and cognitive impairment, whose impact will be even more profound as the global population continues to age (Scheltens et al., 2021; Author Anonymous, 2023). The number of people affected by AD worldwide is estimated to rise from 55 million in 2020 to 135 million by 2050 (Author Anonymous, 2023). The global cost of treating AD is estimated at approximately 2.8 trillion dollars, which will create a huge social burden. The development of safe and effective anti-AD drugs has always been the hot spot in medical chemistry.

The pathogenesis of AD is complex, and many hypotheses were proposed, including the cholinergic hypothesis, the beta-amyloid cascade hypothesis, the oxidative stress hypothesis, the metal ion disorder hypothesis, etc. In the past years, major research institutions and pharmaceutical companies have invested hundreds of billions of dollars in AD. At present, the drugs that have been approved, such as AChE inhibitors and NMDAR inhibitors, only alleviate the symptoms or make them slightly better, and there is no specific drug that can completely cure AD (Cerejeira et al., 2012; Marcinkowska et al., 2021). Therefore, more and more researchers have turned their attention to the multi-target design strategy. Multi-target drugs are expected to become a breakthrough in the treatment of AD (Rossi et al., 2021; Babaei et al., 2022; Turgutalp et al., 2022).

Melatonin (MT) is a neurohormone secreted by the pineal gland (Somalo-Barranco et al., 2022). As an endogenous natural active substance of the human body, accumulating reports suggest that melatonin has excellent antioxidant activity and a protective effect on nerve cells (He et al., 2022). Besides, MT can also chelate heavy metals, including lead, cadmium, and aluminum, while chelating iron and copper can reduce oxidative stress in the body (Reiter et al., 2016). Furthermore, clinical studies have shown that melatonin can improve cognition and mood in Alzheimer’s patients (Dowling et al., 2008). In the meantime, the imbalance of metal ions in the brain will accelerate the aggregation of Aβ and Tau proteins resulting in cognitive decline. Hence, metal ion chelators, such as hydroxyquinoline derivative clioquinol (CQ) are considered potential drugs for the treatment of AD (Adlard et al., 2008; Faux et al., 2010). From this, a hypothesis can be proposed that simultaneously targeting oxidative stress and metal ion disorder may be an effective strategy for treating AD.

**FIGURE 1:**
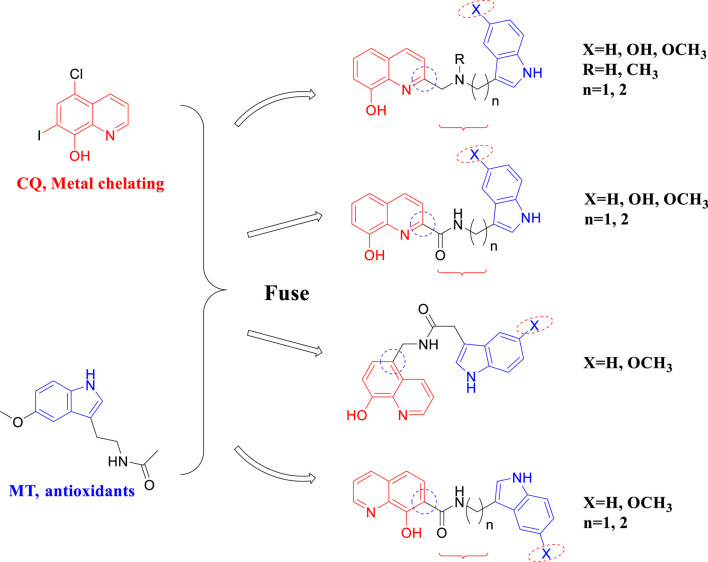
The design strategy of melatonin–hydroxyquinoline hybrids.

In this study, a series of novel melatonin–hydroxyquinoline hybrids were designed and synthesized, targeting anti-oxidation and metal ion chelation at the same time. In detail, melatonin and hydroxyquinoline were coupled by amide or amine linkage, and the connecting linker’s length and the hydroxyquinoline’s link location were investigated for their impact on bioactivity (Figure 1).

## 2 Results and discussion

### 2.1 Synthesis

**SCHEME 1:**
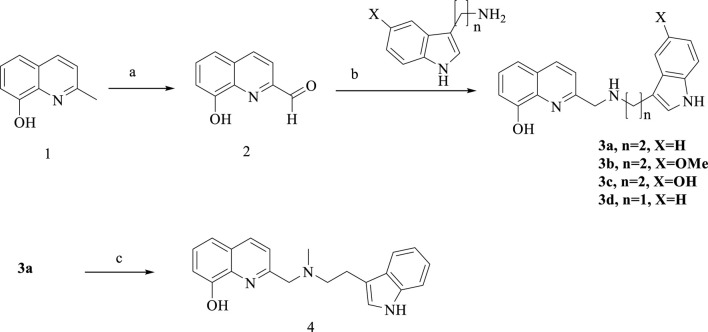
Synthesis of **3a-d** and **4**. Reagent and conditions: (a) SeO_2_/1,4-dioxane, 60°C to reflux; 86%. (b) NaBH_4_, isopropanol, room temperature, overnight, 62%–67%; (c) CH_3_I, K_2_CO_3_, acetone, 60%.

**SCHEME 2:**
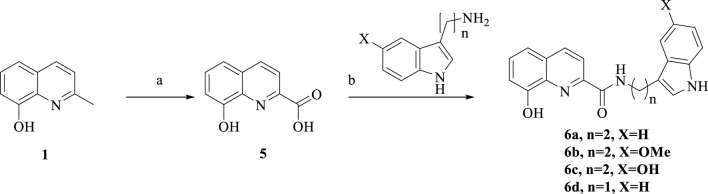
Synthesis of **6a-d**. Reagent and conditions: (a) SeO_2_, pyridine, 120°C, 12 h, 50%; (b) HATU, DIPEA, anhydrous CH_2_Cl_2_, room temperature, overnight, 60%–70%.

**SCHEME 3:**
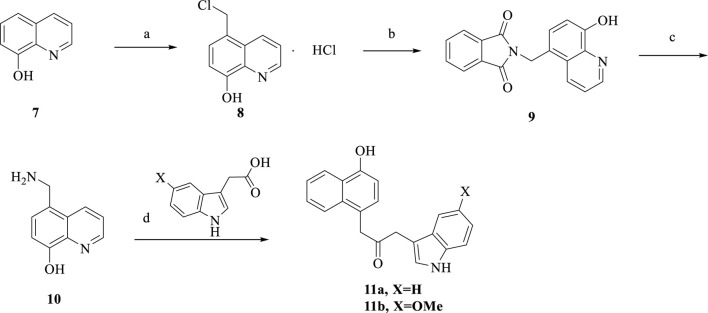
Synthesis of **11a-b**. Reagent and conditions: (a) ZnCl_2_, HCl, formaldehyde, room temperature, 12 h, 85%; (b) DMF, reflux, 8 h, 50%; (c) HCl, reflux, 9 h, 75%; (d) HATU, DIPEA, anhydrous CH_2_Cl_2_, room temperature, overnight, 58%–60%.

**SCHEME 4:**
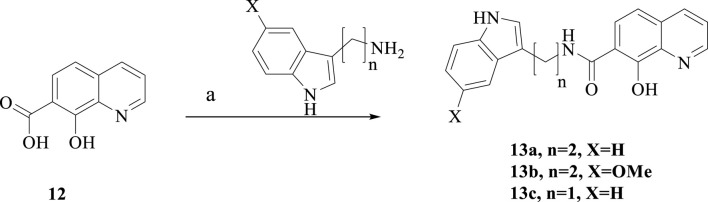
Synthesis of **13a-c**. Reagent and conditions: (a) HATU, DIPEA, anhydrous CH_2_Cl_2_, room temperature, overnight, 40%–55%.

Routes for synthesizing the target compounds (**3a-d**, **4**, **6a-d**, **11a-b** and **13a-c**) are demonstrated in Schemes 1–4. Commercially available 2-methyl-8-hydroxyquinoline (**1**) was reacted with SeO_2_ in the presence of 1,4-dioxane to produce compound **2**. Dissolving different amines, compound **2** and isopropanol, they were reacted at room temperature for 3 h, then NaBH_4_ was added and kept stirring for 12 h to obtain **3a-d**. Target compound **4** was obtained by the *N*-methylationst of **3a**.

The synthetic route of **6a-d** *via* a tow-step produce is shown in Scheme 2. Commercially available 2-methyl-8-hydroxyquinoline was reacted with SeO_2_ in pyridine to produce compound **5**, followed by treatment with amines, HATU and DIPEA to mediate amine formation, so **6a-d** were obtained.

The synthesis of the target compounds **11a-b** is summarized in Scheme 3. Using ZnCl_2_ as a catalyst, 8-hydroxyquinoline was reacted with formaldehyde and hydrochloric acid to produce **8**. Compound **8** was refluxed in DMF for 8 h. Then refluxed in hydrochloric acid for 9 h to obtain compound **10**. Followed by treatment with different substituted carboxylic acids, HATU and DIPEA to produce target **11a-b**.

As showed in Scheme 4, treatment of the commercially available 8-hydroxyquinoline-7-carboxylic acid and different amines in the presence of HATU and DIPEA afforded the target compounds **13a-b**.

### 2.2 Activity evaluation and structure-activity relationships

#### 2.2.1 The oxygen radical absorbance capacity (ORAC)

Oxidative stress is involved in various pathological processes of AD and is one of the crucial adjective pathogeneses of AD. Therefore, it is essential to test the scavenging ability of target compounds. The oxygen radical absorbance capacity (ORAC) (Wang et al., 2014; Wang et al., 2015) was tested to evaluate the effects of compounds on oxidative stress. As shown in Table 1, MT has a satisfactory antioxidant effect, with the ORAC values of 2.38, whereas CQ had almost no antioxidant effect. Compared with the melatonin, most of the target compounds, such as 3a∼3d, 4, 6a∼6d, and 11a∼11b, which ORAC values were greater than 2.3. while compound **13a∼13c**, which is linked at position **7** of hydroxyquinoline, have weaker oxygen radical scavenging ability. This suggests that the position of the junction on CQ has a large effect on the activity. Furthermore, the change in the connecting linker’s length has a slight effect on the ORAC, just as 3a and 3d have different lengths while having similar antioxidant activity.

**TABLE 1:** Oxygen radical absorbance capacity, PAMPA assay and inhibition of Aβ_1-42_ self-aggregation for target compounds.

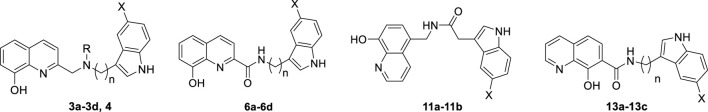							
Compound	n	X	R	ORAC^a^	Inhibition of Aβ_1–42_ aggregation (%)^b^	P_e_ (× 10^–6^ cm s^−1^)^c^	pred
3a	2	H	H	3.50 ± 0.12	27.10 ± 5.01	8.08 ± 0.56	CNS+
3b	2	OMe	H	3.25 ± 0.10	18.23 ± 2.02	6.61 ± 0.22	CNS+
3c	2	OH	H	4.47 ± 0.18	40.23 ± 2.89	4.81 ± 0.87	CNS+
3d	1	H	H	2.60 ± 0.20	20.47 ± 4.72	5.09 ± 1.32	CNS+
4	2	H	Me	2.93 ± 0.15	52.54 ± 6.47	9.87 ± 0.66	CNS+
6a	2	H	—	2.49 ± 0.22	45.45 ± 0.41	7.71 ± 0.61	CNS+
6b	2	OMe	—	2.76 ± 0.01	63.24 ± 2.02	6.91 ± 0.92	CNS+
6c	2	OH	—	3.23 ± 0.02	40.33 ± 2.09	2.96 ± 0.58	CNS±
6d	1	H	—	2.32 ± 0.17	19.25 ± 5.85	6.81 ± 1.13	CNS+
11a	2	H	—	2.91 ± 0.16	44.74 ± 5.18	2.82 ± 0.42	CNS±
11b	2	OMe	—	3.58 ± 0.12	34.94 ± 2.91	1.35 ± 0.25	CNS−
13a	2	H	**—**	1.06 ± 0.07	46.98 ± 6.25	3.98 ± 0.45	CNS±
13b	2	OMe	—	1.33 ± 0.02	19.66 ± 2.06	1.12 ± 0.42	CNS−
13c	1	H	—	0.94 ± 0.02	38.58 ± 5.12	2.65 ± 0.46	CNS±
CQ	—	—		0.54 ± 0.17	30.76 ± 1.08	NT	NT
Melatonin	—	—		2.38 ± 0.12	38.96 ± 9.35	NT	NT
Curcumin	—	—		NT	52.88 ± 6.38	NT	NT
Chlorpromazinne	—	—		NT	NT	6.63 ± 0.81	CNS+
Hydrocortisone	—	—		NT	NT	1.13 ± 0.15	CNS−

^a^ Result is the mean of three independent experiments (*n* = 3) ± SD.

^b^ Result are the mean of three independent experiments (*n* = 3) ± SD., The concentration of all compounds was 20 μM.

^c^ Result are the mean of three independent experiments (*n* = 3) ± SD., Compounds could potentially cross the BBB, when Pe > 4.7 × 10−6 cm s−1.

NT, not tested.

#### 2.2.2 Inhibition of Aβ_1–42_ aggregation

Self-mediated Aβ1–42 aggregation inhibition was assessed via thioflavin T (ThT) fluorescence assay (Rosini et al., 2008; Wang et al., 2018; Babaei et al., 2022). As shown in Table 1, both CQ and MT showed good anti-aggregation effect. Most of the target compounds showed good inhibition of Aβ_1-42_ aggregation. Among them, **3c**, **4**, **6a**, **6b**, **6c**, **11a**, and **13a** showed better activity than melatonin (38.96 ± 9.35) and CQ (30.76 ± 1.08). When the connection is amine, compounds with -OH substituents on the indole ring have the highest inhibitory activity against Aβ_1–42_ aggregation. Compound **3c** containing hydroxyl-substituted indole ring fragments has the highest inhibitory rate against Aβ_1-42_ self-aggregation (40.23%), which is much higher than the inhibition rate of methoxy-substituted and unsubstituted indole ring fragment compounds (18.23% and 27.10% respectively). When the connection mode is an amide, compound **6b** with a methoxy group on the indole ring has the highest inhibition rate (63.24%). This shows that the different substituents at position 5 on the indole ring and the different link methods between melatonin and hydroxyquinoline play an important role in inhibiting the self-aggregation of Aβ_1-42_. Compared **3a** (27.10%) with **4** (52.54%), it can be found that methylation of nitrogen atoms leads to increased activity. On the whole when the connection mode is amide, the inhibitory activity is better. The length of the connected chain is shortened, and the activity decreases to different degrees. In addition, the compounds obtained at positions 2, 5 and 7 of the quinoline ring had no significant effect on the self-aggregation of Aβ_1-42_ such as **6a** (45.45%), **11a** (44.74%) and **13a** (46.98%).

#### 2.2.3 Blood–brain barrier permeability assay

The blood-brain barrier permeability of central nervous drugs is a crucial drug-like property. In this work, the parallel artificial membrane permeability assay (PAMPA) (Di et al., 2003; Wang et al., 2014) was used to evaluate the ability of compounds to cross the blood-brain barrier. 13 commercial drugs were chosen to establish the evaluation system (Supplementary Table S1). Most of the target compounds can cross the blood-brain barrier with the *P*_*e*_ > 4.7, such as **3a**, **3b**, **3c**, **3d**, **4**, **6a, 6b** and **6d**. The compounds with hydroxyl groups exhibit poor BBB permeability, possibly due to increased hydrophilia.

#### 2.2.4 Cytotoxicity assay

**FIGURE 2:**
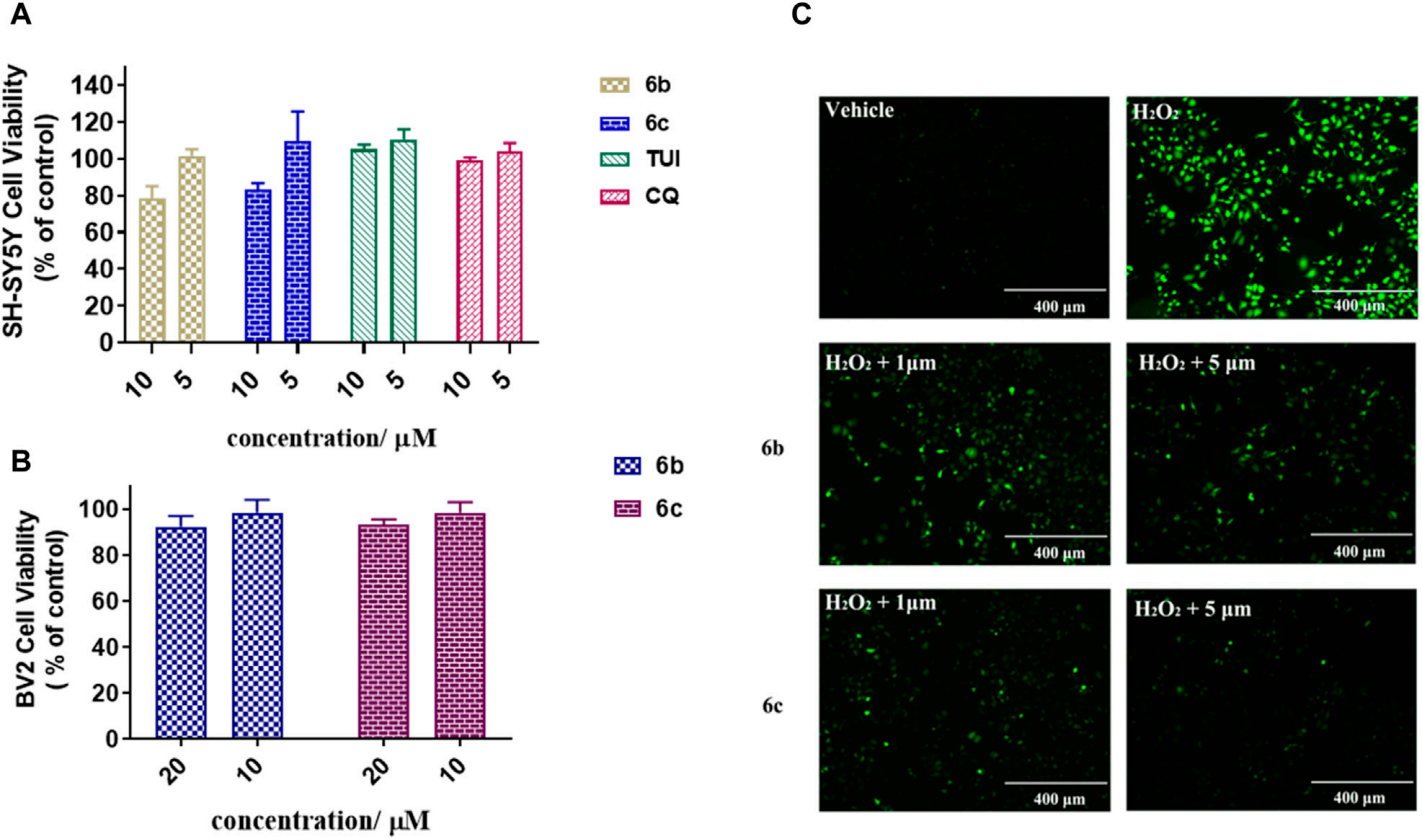
Cytotoxicity assay of 6b and 6c on SH-SY5Y **(A)** and BV2 **(B)**. Protective effect of **6b** and **6c** against H_2_O_2_ induced SH-SY5Y oxidative stress **(C)**.

To further investigate the bioactivity of the compounds, cytotoxicity was evaluated on SH-SY5Y and BV2 cell lines (Figures 2A, B). The results showed that **6b** and **6c** exhibit no cytotoxicity at 5 μM in SH-SY5H cell lines while showing slight toxicity at concentrations of 10 μM. As for BV2 cells, **6b** and **6c** also showed no significant cytotoxicity at 20 μM concentration.

#### 2.2.5 Alleviating oxidative stress induced by hydrogen peroxide

The effect of **6b** and **6c** on the oxidative stress of SH-SY5H cells induced by hydrogen peroxide was investigated using DCFH-DA as the fluorescent probe (Xu et al., 2021). The results showed that the ROS increased sharply in SH-SY5H cells treated with 400 μM hydrogen peroxide for 12 h. Pretreatment with **6b** or **6c** reduced ROS production in a dose-dependent manner, suggesting that **6b** and **6c** have a good ability to alleviate oxidative stress (Figure 2C).

#### 2.2.6 Metal-chelating property

**FIGURE 3:**
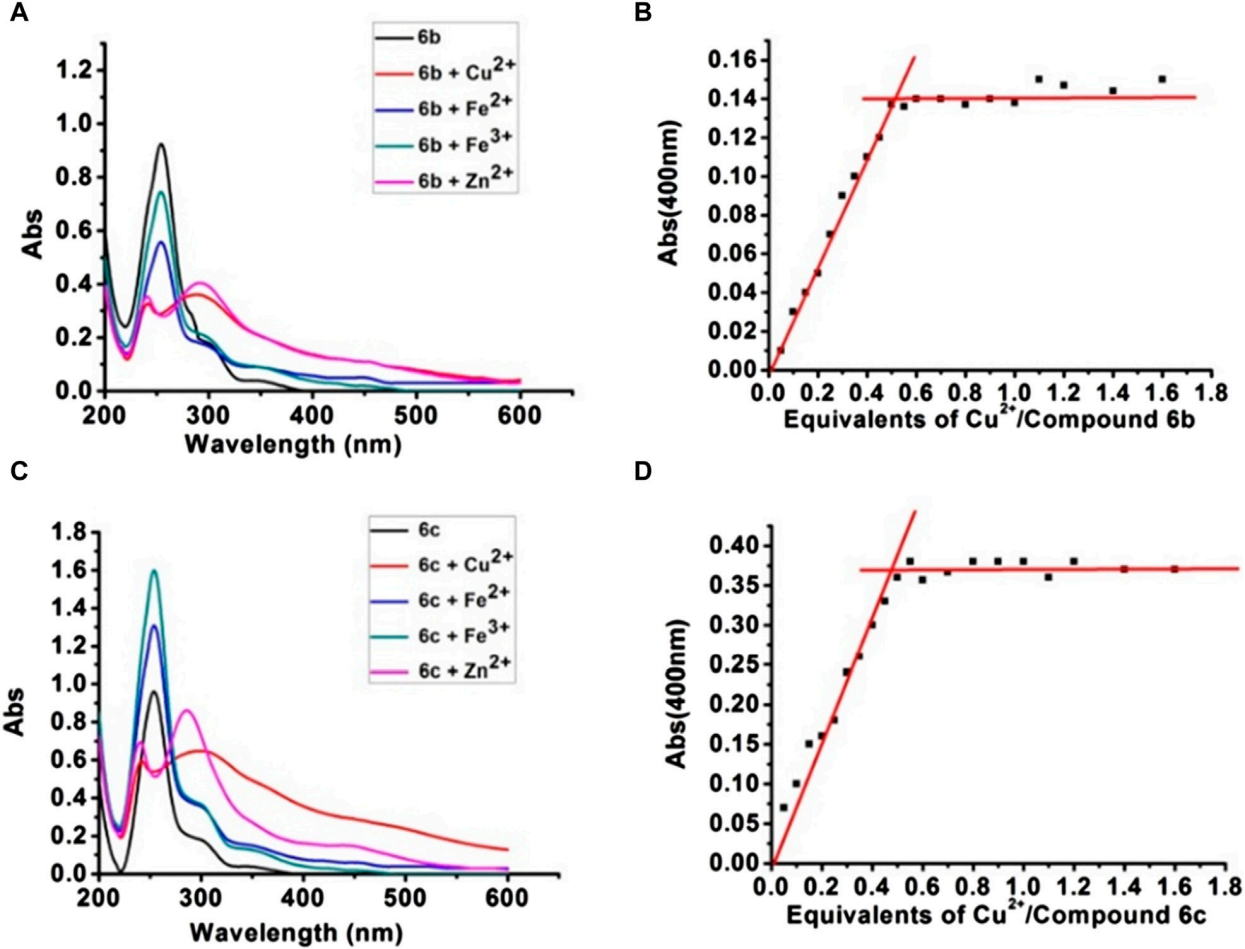
UV-vis absorption spectra of compounds **6b**, **6c** with Cu^2+^, Zn^2+^, Fe^2+^, and Fe^3+^ **(A, C)**. The chelation ratios of **6b** and **6c** to Cu^2+^ **(B, D)**.

To further evaluate multi-target anti-AD potential, the metal-chelating properties of **6b** and **6c** were determined by UV spectrophotometry (Geng et al., 2012; Lu et al., 2013; Hu et al., 2019). As shown in Figures 3A, C, **6b** exhibited a maximum absorption peak at 255 nm, and the maximum absorption peak showed a significant redshift and the intensity decreased when **6b** co-incubated with Cu^2+^ or Zn^2+^. While co-incubated with Fe^3+^ or Fe^2+^, the intensity at 255 nm was in different degrees decreased. The same result occurred for **6c**. Those results suggested that **6b** and **6c** possess metal-chelating properties. Next, the chelation ratios of **6b** and **6c** to Cu^2+^ were measured and calculated using the inflection point method (Figures 3B, D). The inflection point appears when the concentration ratio of compound Cu^2+^ to **6b** or **6c** is 0.5, so it can be inferred that the chelation ratio of compound **6b** and **6c** to Cu^2+^ is 2:1.

#### 2.2.7 Effect of Cu^2+^-induced Aβ aggregation

**FIGURE 4:**
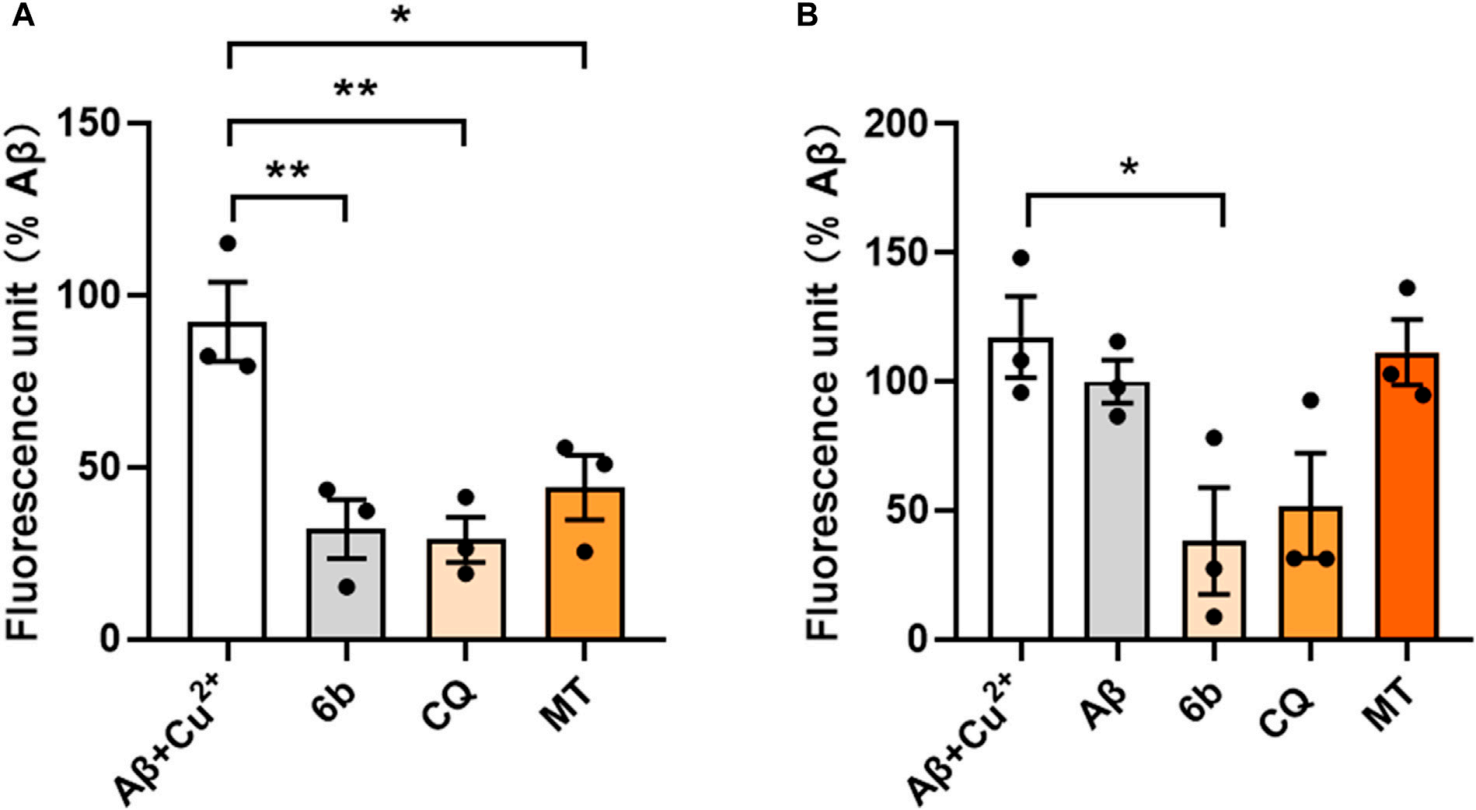
Thioflavin T fluorometric detection. Compounds were incubated before **(A)** or after **(B)** the pre-fibrillation of Aβ_1-42_ and Cu^2+^ at 37°C for 24 h.

To further detect the ability of compounds **6b**, CQ and MT to disaggregate or inhibit the Cu^2+^-induced Aβ_1-42_ aggregation, we conducted thioflavin T fluorometric detection (Hu et al., 2019). The results showed that compounds **6b** and CQ both significantly disaggregated Cu^2+^-induced Aβ_1-42_ aggregation, also with the mild disaggregated effect of MT (Figure 4A). As shown in Figure 4B, **6b** exhibited a distinct inhibitory effect on Cu^2+^-induced Aβ_1-42_ aggregation, which was superior to the reference compound CQ, while MT exhibited weak inhibitory activity. Those results suggest that **6b** can significantly mitigate metal ion induced Aβ aggregation.

## 3 Experimental

### 3.1 Chemistry

#### 3.1.1 8-hydroxyquinoline-2-carbaldehyde (**2**)

At 60°C, 10 mL dioxane solution of 2-methyl-8-hydroxyquinoline (10 mmol) was added to 50 mL dioxane solution of SeO_2_ (20 mmol). After half an hour of drip adding, the reaction reflux for 4 h. Yield 86%. ^1^H NMR (400 MHz, Acetone-*d*_*6*_) *δ* 10.16 (s, 1H), 9.22 (s, 1H), 8.55 (d, *J =* 8.5 Hz, 1H), 8.04 (d, *J =* 8.5 Hz, 1H), 7.69 (t, *J =* 8.0 Hz, 1H), 7.57 (dd, *J =* 8.2, 0.9 Hz, 1H), 7.28 (dd, *J =* 7.7, 1.0 Hz, 1H).

#### 3.1.2 8-hydroxyquinoline-2-carboxylic acid (5)

**1** (20 mmol) was dissolved in pyridine (50 mL), and SeO_2_ (20 mmol) was added followed by stirring at 120°C for 12 h, after the completion of the reaction (monitored by TLC). The reaction mixture was filtered off first. The solvent was distilled off and the residue was dissolved in an aqueous KOH solution (10%), Then filtered and the filtered liquid was acidified with hydrochloric acid (10%). Last the filtered crude product was purified by silica-column chromatography. yellow solid, yield 50%.^1^H NMR (400 MHz, Acetone-*d*_*6*_) *δ* 9.68 (s, 1H), 8.63 (d, *J =* 8.5 Hz, 1H), 8.27 (d, *J =* 8.5 Hz, 1H), 7.70 (t, *J =* 7.9 Hz, 1H), 7.60 (d, *J =* 8.2 Hz, 1H), 7.28 (d, *J =* 7.6 Hz, 1H).

#### 3.1.3 5-(Chloromethyl)quinolin-8-ol hydrochloride (8·HCl)

Compound **8** was synthesized according to the literature. Pale yellow solid, yield 98%. ^1^H NMR (400 MHz, DMSO-*d*_6_) *δ* 9.22 (d, *J =* 7.9 Hz, 1H), 9.13 (dd, *J =* 5.0, 1.1 Hz, 1H), 8.12 (dd, *J =* 8.6, 5.1 Hz, 1H), 7.87 (d, *J =* 8.0 Hz, 1H), 7.51 (d, *J =* 8.0 Hz, 1H), 5.33 (s, 2H).

#### 3.1.4 2-(hydroxyquinoline-5-yl-methyl)-isoquinoline-1,3-diketone (9)

Under the nitrogen atmosphere. A mixture of phthalimide potassium (4.5 mmol), 5-(Chloromethyl)quinolin-8-ol Hydrochloride (3 mmol) and DMF (10 mL) was heated to 150°C, and refluxed for 8 h. After cooling to room temperature, the white potassium chloride residue was formed at the bottom of the flask, which was filtered. The filtrate was poured into water (400 mL), and filtered to obtain compound **9**. White solid, yields 73%. ^1^H NMR (400 MHz, DMSO-*d*_6_) *δ* 9.82 (s, 1H), 8.88 (dd, *J =* 4.1, 1.2 Hz, 1H), 8.73 (dd, *J =* 8.6, 1.3 Hz, 1H), 7.92–7.87 (m, 2H), 7.87–7.82 (m, 2H), 7.65 (dd, *J =* 8.6, 4.1 Hz, 1H), 7.45 (d, *J =* 7.9 Hz, 1H), 7.03 (d, *J =* 7.9 Hz, 1H), 5.14 (s, 2H).

#### 3.1.5 5-aminomethyl-8-hydroxyquinoline (10)

To a stirred concentrated hydrochloric acid (20 mL), compound **9** (2.19 mmol) was added, refluxed for 9 h until the mixture became transparent after cooling to room temperature. The solvent was distilled off and the residue was dissolved in water, then the solution pH to produce a solid. Greenish solid, yield 75%. ^1^H NMR (400 MHz, DMSO-*d*_6_) *δ* 8.85 (dd, *J =* 4.1, 1.4 Hz, 1H), 8.56 (dd, *J =* 8.5, 1.4 Hz, 1H), 7.57 (dd, *J =* 8.5, 4.1 Hz, 1H), 7.42 (d, *J =* 7.8 Hz, 1H), 7.01 (d, *J =* 7.8 Hz, 1H), 4.08 (s, 2H).

##### 3.1.5.1 General method for the preparation of compounds 3a-3d

To a solution of **2** (1 mmol) and different indole (1 mmol) in isopropyl alcohol. After stirring at room temperature for 3 h, the NaBH_4_ (2 mmol) was added and kept stirring for 12 h. Quenched with water, extracted with ethyl acetate, the organic layer was dried with Na_2_SO_4_ and concentrated under reduced pressure. The residue was purified by silica gel chromatography to afford **3a**-**3d** (CH_2_Cl_2_/CH_3_OH = 50:1).

#### 3.1.6 2-(((2-(1H-indol-3-yl)ethyl)amino)methyl)quinolin-8-ol (3a)

Yellow solid, yield 65%. ^1^H NMR (400 MHz, DMSO-*d*_6_) *δ* 10.76 (s, 1H), 8.24 (d, *J =* 8.5 Hz, 1H), 7.56 (d, *J =* 8.5 Hz, 1H), 7.51 (d, *J =* 7.8 Hz, 1H), 7.42–7.37 (m, 1H), 7.37–7.33 (m, 1H), 7.32 (d, *J =* 8.1 Hz, 1H), 7.14 (d, *J =* 1.8 Hz, 1H), 7.09–7.06 (m, 1H), 7.06–7.01 (m, 1H), 6.94 (t, *J =* 7.4 Hz, 1H), 4.07 (s, 2H), 2.91 (s, 2H), 2.90 (s, 2H). ^13^C NMR (126 MHz, DMSO-*d*_6_) *δ* 159.23, 153.23, 137.91, 136.69, 136.66, 128.05, 127.75, 127.23, 122.96, 121.45, 121.28, 118.76, 118.58, 117.98, 113.10, 111.79, 111.46, 55.13, 50.53, 26.04. HRMS (ESI) m/z calcd for C_20_H_19_N_3_O [M + H]^+^, 318.1562; found, 318.1562. HPLC purity: 99.6%, retention time: 8.8 min.

#### 3.1.7 2-(((2-(5-methoxy-1H-indol-3-yl)ethyl)amino)methyl)quinolin-8-ol (3b)

Yellow solid, yield 67%. ^1^H NMR (400 MHz, DMSO-*d*_6_) *δ* 10.61 (s, 1H), 8.25 (d, *J =* 8.5 Hz, 1H), 7.57 (d, *J =* 8.5 Hz, 1H), 7.42–7.37 (m, 1H), 7.37–7.31 (m, 1H), 7.20 (d, *J =* 8.7 Hz, 1H), 7.10 (d, *J =* 2.1 Hz, 1H), 7.07 (dd, *J =* 7.0, 1.6 Hz, 1H), 6.94 (d, *J =* 2.2 Hz, 1H), 6.68 (dd, *J =* 8.7, 2.3 Hz, 1H), 4.08 (s, 2H), 3.68 (s, 3H), 2.88 (s, 4H). ^13^C NMR (126 MHz, DMSO-*d*_6_) *δ* 159.19, 153.34, 153.24, 137.91, 136.66, 131.83, 128.05, 128.01, 127.24, 123.67, 121.45, 117.97, 112.85, 112.45, 111.48, 111.46, 100.47, 55.70, 55.06, 50.38, 26.05. HRMS (ESI) m/z calcd for C_21_H_21_N_3_O_2_ [M + H]^+^, 348.1678; found,348.1667. HPLC purity: 98.6%. Retention time: 8.7 min.

#### 3.1.8 2-(((2-(5-hydroxy-1H-indol-3-yl)ethyl)amino)methyl)quinolin-8-ol (3c)

Yellow solid, yield 62%. ^1^H NMR (400 MHz, DMSO-*d*_6_) *δ* 10.45 (s, 1H), 8.26 (d, *J =* 8.5 Hz, 1H), 7.57 (d, *J =* 8.5 Hz, 1H), 7.45–7.38 (m, 1H), 7.38–7.33 (m, 1H), 7.12 (d, *J =* 8.6 Hz, 1H), 7.08 (dd, *J =* 7.0, 1.4 Hz, 1H), 7.04 (d, *J =* 1.7 Hz, 1H), 6.83 (d, *J =* 1.9 Hz, 1H), 6.58 (dd, *J =* 8.6, 2.1 Hz, 1H), 4.10 (s, 2H), 3.00 (d, J = 6.8 Hz, s), 2.85 (d, *J =* 6.6 Hz, 2H). ^13^C NMR (126 MHz, DMSO-*d*_6_) *δ* 158.84, 153.22, 150.59, 137.88, 136.73, 131.29, 128.42, 128.06, 127.29, 123.46, 121.40, 118.00, 112.11, 111.96, 111.68, 111.51, 102.73, 55.01, 50.37, 26.01. HRMS (ESI) m/z calcd for C_20_H_19_N_3_O_2_ [M + H]^+^, 348.1678; found,348.1667. HPLC purity: 98.1%. Retention time: 8.4 min.

#### 3.1.9 2-((((1H-indol-3-yl)methyl)amino)methyl)quinolin-8-ol (3d)

Yellow solid, yield 64%. ^1^H NMR (400 MHz, DMSO-*d*_6_) *δ* 10.90 (s, 1H), 8.25 (d, *J =* 8.5 Hz, 1H), 7.65 (d, *J =* 7.8 Hz, 1H), 7.58 (d, *J =* 8.5 Hz, 1H), 7.41–7.38 (m, 1H), 7.38–7.36 (m, 1H), 7.36–7.34 (m, 1H), 7.30 (s, 1H), 7.10–7.08 (m, 1H), 7.08–7.06 (m, 1H), 6.97 (t, J = 7.2 Hz, 1H), 4.07 (s, 2H), 3.99 (s, 2H). ^13^C NMR (126 MHz, DMSO-*d*_6_) *δ* 158.77, 153.24, 137.89, 136.82, 136.68, 128.06, 127.52, 127.26, 124.32, 121.49, 121.43, 119.34, 118.79, 117.98, 113.49, 111.80, 111.50, 54.35, 44.43. HRMS (ESI) m/z calcd for C_19_H_17_N_3_O [M + H]^+^, 304.1423; found,304.1405. HPLC purity: 98. Retention time: 13.7 min.

#### 3.1.10 2-(((2-(1H-indol-3-yl)ethyl)(methyl)amino)methyl)quinolin-8-ol (4)

To a solution of **3a** (0.5 mmol) and K_2_CO_3_ (1 mmol) in acetone, CH_3_I (0.5 mmol) was added slowly. After being stirred at room temperature overnight, the solvent was evaporated, followed by extraction with CH_2_Cl_2_. The combined organic layer was dried over anhydrous Na_2_SO_4_ and concentrated under reduced pressure. The residue was purified by silica gel chromatography to afford yellow oil (CH_2_Cl_2_/CH_3_OH = 30: 1). Yellow solid, yield 66%. ^1^H NMR (400 MHz, MeOD) *δ* 8.16 (d, *J =* 8.5 Hz, 1H), 7.44 (d, *J =* 8.4 Hz, 1H), 7.41 (s, 1H), 7.39 (s, 1H), 7.33 (d, *J =* 7.6 Hz, 1H), 7.29 (d, *J =* 8.1 Hz, 1H), 7.10 (d, *J =* 7.5 Hz, 1H), 7.04 (d, *J =* 7.6 Hz, 1H), 7.02 (s, 1H), 6.88 (t, *J =* 7.5 Hz, 1H), 4.00 (s, 2H), 3.06 (dd, *J =* 9.8, 6.4 Hz, 2H), 2.87 (dd, *J =* 9.7, 6.4 Hz, 2H), 2.50 (s, 3H).^13^C NMR (126 MHz, MeOD) *δ* 156.15, 153.00, 138.01, 136.73, 136.46, 128.00, 127.22, 127.04, 121.74, 121.40, 120.87, 118.10, 117.80, 117.40, 112.18, 110.83, 110.66, 62.75, 58.27, 41.63, 22.31. HRMS (ESI) m/z calcd for C_21_H_21_N_3_O [M + H]^+^, 332.1732; found, 332.1718. HPLC purity: 97.8%. Retention time: 11.6 min.

##### 3.1.10.1 General method for the preparation of compounds 6a-6d, 11a-11b and 13a-13c

To a solution of quinoline acid or quinoline amine (1 mmol) and indole amine or indole acid (1.3 mmol) in anhydrous CH_2_Cl_2,_ HATU (1 mmol) and DIPEA (2 mmol) were added. After stirred at room temperature overnight, the reaction was extracted with CH_2_Cl_2_. The combined organic phase was dried and evaporated, the target compounds were purified by column chromatography *via* CH_2_Cl_2_/MeOH mixture. (CH_2_Cl_2_: MeOH = 50:1.)

#### 3.1.11 N-(2-(1H-indol-3-yl) ethyl)-8-hydroxyquinoline-2-carboxamide (6a)

Pale yellow solid, yield 60%. ^1^H NMR (500 MHz, DMSO-*d*_6_) *δ* 10.85 (s, 1H), 10.13 (s, 1H), 9.82 (t, *J =* 6.0 Hz, 1H), 8.51 (d, *J =* 8.5 Hz, 1H), 8.17 (d, *J =* 8.5 Hz, 1H), 7.63 (d, *J =* 7.8 Hz, 1H), 7.57 (t, *J =* 7.9 Hz, 1H), 7.52–7.45 (m, 1H), 7.35 (d, *J =* 8.1 Hz, 1H), 7.22 (d, *J =* 2.1 Hz, 1H), 7.18 (dd, *J =* 7.6, 0.9 Hz, 1H), 7.07 (t, *J =* 7.5 Hz, 1H), 6.99 (t, *J =* 7.4 Hz, 1H), 3.69 (dd, *J =* 14.7, 6.6 Hz, 2H), 3.05 (t, *J =* 7.6 Hz, 2H). ^13^C NMR (126 MHz, DMSO-*d*_6_) *δ* 164.11, 154.08, 148.09, 138.19, 136.88, 136.72, 129.92, 129.81, 127.70, 123.15, 121.44, 119.29, 118.75, 118.64, 118.01, 112.22, 111.99, 111.89, 25.90. HRMS (ESI) m/z calcd for C_20_H_17_N_3_O_2_ [M + H]^+^, 332.1732; found,332.1354. HPLC purity: 98.3%. Retention time: 15.4 min.

#### 3.1.12 8-hydroxy-N-(2-(5-methoxy-1H-indol-3-yl)ethyl)quinoline-2-carboxamide (6b)

Pale yellow solid, yield 62%. ^1^H NMR (500 MHz, DMSO-*d*_6_) *δ* 10.69 (s, 1H), 10.15 (s, 1H), 9.83 (s, 1H), 8.50 (d, *J =* 8.3 Hz, 1H), 8.19 (d, *J =* 8.3 Hz, 1H), 7.57 (t, *J =* 7.6 Hz, 1H), 7.49 (d, *J =* 7.8 Hz, 1H), 7.25 (d, *J =* 8.5 Hz, 1H), 7.20 (s, 1H), 7.18 (s, 1H), 7.12 (s, 1H), 6.72 (d, *J =* 8.0 Hz, 1H), 3.72 (s, 3H), 3.69 (s, 2H), 3.04 (s, 2H). ^13^C NMR (126 MHz, DMSO-*d*_6_) *δ* 164.12, 154.08, 153.48, 148.11, 138.17, 136.89, 131.84, 129.91, 129.81, 128.05, 123.83, 119.29, 118.00, 112.52, 112.12, 111.99, 111.63, 100.56, 55.71, 25.90. HRMS (ESI) m/z calcd for C_21_H_19_N_3_O_3_ [M + H]^+^, 362.1471; found, 362.1460. HPLC purity: 99.6%. Retention time: 13.4 min.

#### 3.1.13 8-hydroxy-N-(2-(5-hydroxy-1H-indol-3-yl)ethyl)quinoline-2-carboxamide (6c)

Pale yellow solid, yield 60%. ^1^H NMR (400 MHz, DMSO-*d*_6_) *δ* 10.50 (s, 1H), 10.11 (s, 1H), 9.78 (t, *J =* 5.8 Hz, 1H), 8.60 (s, 1H), 8.51 (d, *J =* 8.5 Hz, 1H), 8.18 (d, *J =* 8.5 Hz, 1H), 7.57 (t, *J =* 7.9 Hz, 1H), 7.49 (d, *J =* 8.0 Hz, 1H), 7.18 (d, *J =* 7.5 Hz, 1H), 7.14 (d, *J =* 8.6 Hz, 1H), 7.11 (d, *J =* 1.8 Hz, 1H), 6.93 (d, *J =* 1.9 Hz, 1H), 6.61 (dd, *J =* 8.6, 2.1 Hz, 1H), 3.65 (dd, *J =* 14.8, 6.6 Hz, 2H), 2.99–2.93 (m, 2H). ^13^C NMR (126 MHz, DMSO-*d*_6_) *δ* 164.09, 154.07, 150.72, 148.12, 138.18, 136.89, 131.30, 129.92, 129.81, 128.37, 123.57, 119.29, 118.01, 112.18, 111.99, 111.82, 111.24, 102.70, 26.03. HRMS (ESI) m/z calcd for C_20_H_17_N_3_O_3_ [M + H]^+^, 348.1313; found, 348.1303. HPLC purity: 99.5%. Retention time: 8.0 min.

#### 3.1.14 N-((1H-indol-3-yl)methyl)-8 -hydroxyquinoline-2-carboxamide (6d)

Pink solid, yield 60%. ^1^H NMR (400 MHz, DMSO-*d*_6_) *δ* 10.96 (s, 1H), 10.14 (s, 1H), 9.89 (t, *J =* 5.9 Hz, 1H), 8.50 (d, *J =* 8.6 Hz, 1H), 8.23 (t, *J =* 8.3 Hz, 1H), 7.65 (d, *J =* 7.9 Hz, 1H), 7.54 (t, *J =* 7.9 Hz, 1H), 7.46 (d, *J =* 8.1 Hz, 1H), 7.39–7.37 (m, 1H), 7.37–7.35 (m, 1H), 7.13 (d, *J =* 7.5 Hz, 1H), 7.07 (t, *J =* 7.5 Hz, 1H), 6.97 (t, *J =* 7.5 Hz, 1H), 4.77 (d, *J =* 5.9 Hz, 2H). ^13^C NMR (126 MHz, DMSO-*d*_6_) *δ* 163.90, 154.13, 148.13, 138.16, 136.91, 136.87, 129.91, 129.80, 126.96, 124.49, 121.67, 119.47, 119.23, 119.07, 117.96, 112.77, 112.01, 111.98, 34.80. HRMS (ESI) m/z calcd for C_19_H_15_N_3_O_2_ [M + H]^+^, 318.1573; found, 318.1198. HPLC purity: 97.9%. Retention time: 16.5 min.

#### 3.1.15 N-((8-hydroxyquinolin-5-yl)methyl)-2-(1H-indol-3-yl)acetamide (11a)

Pale white solid, yield 58%. ^1^H NMR (400 MHz, DMSO-*d*_6_) *δ* 10.83 (s, 1H), 9.72 (s, 1H), 8.85 (d, *J =* 3.8 Hz, 1H), 8.43 (d, *J =* 8.6 Hz, 1H), 8.35 (t, J = 5.1 Hz, 1H), 7.53–7.48 (m, 1H), 7.48–7.43 (m, 1H), 7.37 (d, *J =* 7.8 Hz, 1H), 7.32 (d, *J =* 8.1 Hz, 1H), 7.15 (s, 1H), 7.05 (t, *J =* 7.5 Hz, 1H), 6.99 (d, *J =* 7.7 Hz, 1H), 6.90 (t, *J =* 7.4 Hz, 1H), 4.62 (d, *J =* 5.5 Hz, 2H), 3.54 (s, 2H). ^13^C NMR (126 MHz, DMSO-*d*_6_) *δ* 170.93, 153.21, 148.24, 139.12, 136.56, 133.23, 128.21, 127.66, 127.30, 125.53, 124.24, 122.14, 121.38, 119.19, 118.65, 111.74, 110.66, 109.32, 54.05, 33.16. HRMS (ESI) m/z calcd for C_20_H_17_N_3_O_2_ [M + H]^+^, 332.1364; found, 332.1354. HPLC purity: 95.2%. Retention time: 8.2 min.

#### 3.1.16 N-((8-hydroxyquinolin-5-yl)methyl)-2-(5-methoxy-1H-indol-3-yl)acetamide (11b)

Pale white solid, yield 60%. ^1^H NMR (400 MHz, DMSO-*d*_6_) *δ* 10.68 (s, 1H), 9.71 (s, 1H), 8.84 (dd, *J =* 4.0, 1.3 Hz, 1H), 8.42 (dd, *J =* 8.5, 1.2 Hz, 1H), 8.35 (t, *J =* 5.3 Hz, 1H), 7.47 (dd, *J =* 8.6, 4.1 Hz, 1H), 7.38 (d, *J =* 7.8 Hz, 1H), 7.22 (d, *J =* 8.7 Hz, 1H), 7.12 (d, *J =* 2.1 Hz, 1H), 6.99 (d, *J =* 5.7 Hz, 1H), 6.98 (s, 1H), 6.70 (dd, *J =* 8.7, 2.4 Hz, 1H), 4.63 (d, *J =* 5.6 Hz, 2H), 3.62 (s, 3H), 3.51 (s, 2H). ^13^C NMR (126 MHz, MeOD) *δ* 173.08, 153.64, 152.64, 147.53, 138.78, 132.55, 131.89, 127.87, 127.29, 127.08, 124.42, 124.33, 121.35, 111.63, 111.50, 109.58, 107.81, 99.89, 54.63, 40.20, 32.85. HRMS (ESI) m/z calcd for C_21_H_19_N_3_O_3_ [M + H]^+^, 362.1469; found, 362.1460. HPLC purity: 96.2%. Retention time: 7.5 min.

#### 3.1.17 N-(2-(1H-indol-3-yl)ethyl)-8 -hydroxyquinoline-7-carboxamide (13a)

Orange solid, yield 40%. ^1^H NMR (400 MHz, DMSO-*d*_6_) *δ* 10.83 (s, 1H), 9.01 (t, *J =* 5.2 Hz, 1H), 8.92 (d, *J =* 3.0 Hz, 1H), 8.34 (d, *J =* 8.2 Hz, 1H), 7.99 (d, *J =* 8.8 Hz, 1H), 7.67–7.63 (m, 1H), 7.62 (d, *J =* 8.6 Hz, 1H), 7.42 (d, *J =* 8.8 Hz, 1H), 7.35 (d, *J =* 8.0 Hz, 1H), 7.22 (s, 1H), 7.08 (t, *J =* 7.4 Hz, 1H), 6.99 (t, *J =* 7.3 Hz, 1H), 3.66 (dd, *J =* 13.4, 6.9 Hz, 2H), 3.03 (t, *J =* 7.3 Hz, 2H). ^13^C NMR (126 MHz, DMSO-*d*_6_) *δ* 168.62, 157.48, 149.58, 139.71, 136.75, 136.45, 131.12, 130.11, 127.68, 125.38, 123.95, 123.27, 121.45, 118.77, 117.35, 112.96, 112.08, 111.89, 40.60, 25.51. HRMS (ESI) m/z calcd for C_20_H_17_N_3_O_2_ [M + H]^+^, 332.1373; found, 332.1354. HPLC purity: 98.9%. Retention time: 5.0 min.

#### 3.1.18 8-hydroxy-N-(2-(5-methoxy-1H-indol-3-yl)ethyl)quinoline-7-carboxamide (13b)

Orange solid, yield 45%. ^1^H NMR (400 MHz, DMSO-*d*_6_) *δ* 10.67 (s, 1H), 9.00 (t, *J =* 5.4 Hz, 1H), 8.92 (dd, *J =* 4.1, 1.5 Hz, 1H), 8.35 (dd, *J =* 8.3, 1.4 Hz, 1H), 8.00 (d, *J =* 8.8 Hz, 1H), 7.65 (dd, *J =* 8.3, 4.2 Hz, 1H), 7.42 (d, *J =* 8.8 Hz, 1H), 7.24 (d, *J =* 8.7 Hz, 1H), 7.18 (d, *J =* 2.1 Hz, 1H), 7.09 (d, *J =* 2.2 Hz, 1H), 6.72 (dd, *J =* 8.7, 2.3 Hz, 1H), 3.66 (dd, *J =* 13.2, 7.0 Hz, 2H), 3.00 (t, *J =* 7.3 Hz, 2H). ^13^C NMR (126 MHz, DMSO-*d*_6_) *δ* 168.62, 157.44, 153.48, 149.58, 139.67, 136.47, 131.87, 131.11, 128.03, 125.38, 123.96, 123.93, 117.35, 112.96, 112.53, 111.93, 111.62, 100.58, 55.71, 40.61, 25.48. HRMS (ESI) m/z calcd for C_21_H_19_N_3_O_3_ [M + H]^+^, 362.1466; found, 362.1460. HPLC purity: 97.5%. Retention time: 5.1 min.

#### 3.1.19 N-((1H-indol-3-yl)methyl) -8-hydroxyquinoline-7-carboxamide (13c)

Orange solid, yield 50%. ^1^H NMR (400 MHz, DMSO-*d*_6_) *δ* 11.00 (s, 1H), 9.12 (t, *J =* 5.2 Hz, 1H), 8.90 (dd, *J =* 4.1, 1.4 Hz, 1H), 8.34 (dd, *J =* 8.3, 1.4 Hz, 1H), 8.06 (d, *J =* 8.8 Hz, 1H), 7.70–7.65 (m, 1H), 7.65–7.62 (m, 1H), 7.41 (d, *J =* 8.8 Hz, 1H), 7.41–7.38 (m, 1H), 7.38–7.36 (m, 1H), 4.75 (d, *J =* 5.4 Hz, 2H). ^13^C NMR (126 MHz, DMSO-*d*_6_) *δ* 167.89, 157.00, 149.47, 139.58, 136.81, 136.51, 131.06, 126.94, 125.77, 124.75, 123.93, 121.70, 119.17, 119.10, 117.36, 113.23, 112.19, 112.01, 35.04. HRMS (ESI) m/z calcd for C_19_H_15_N_3_O_2_ [M + H]^+^, 318.1219; found, 318.1198. HPLC purity: 99.5%. Retention time: 4.7 min.

### 3.2 Thioflavin T fluorometric detection

Detection of the compounds for disaggregated and inhibitory effects on Cu^2+^ induced Aβ_1-42_ aggregation. Aβ_1-42_ (Sigma-Aldrich A9810, 20 μM) was dissolved in HEPES buffer (pH = 6.6, containing 1% ammonium hydroxide). 10 μL of Aβ_1-42_ was previously incubated with or without Cu^2+^ at 37°C for 3 days to pre-fibrillation. 10 μL of **6b**, CQ, and MT (150 μM, in DMSO) were incubated before or after the pre-fibrillation of Aβ_1-42_ at 37°C for 1 day. After incubation, 170 μL of thioflavin T (5 μM, in 50 mM glycine-NaOH buffer) was added to mix well. After incubation for 5 min, the Aβ_1-42_ aggregation was detected by Microplate Reader (HITACHI, F-4700) with excitation/emission at 450/485 nm.

#### 3.2.1 Oxygen radical absorbance capacity (ORAC-FL) assay

The testes compounds and fluorescein (FL) stock solution were diluted with 75 mM phosphate buffer (pH 7.4) to 5 µM and 0.117 µM, respectively (Wang et al., 2018). The solution of Trolox was diluted with 75 mM phosphate buffer to 40, 20, 10, 5, 2.5, and 1.25 µM. The solution of 2,2′-azobis- (amidinopropane) dihydrochloride (AAPH) was prepared to a final concentration of 40 mM. The mixture of the tested compounds (20 µL) and FL (120 μL; 70 nM) was pre-incubated for 10 min at 37°C, 60 µL of the AAPH solution was added. The fluorescence was recorded every minute for 120 min (excitation, 485 nm; emission, 520 nm). The antioxidant curves (fluorescence versus time) were normalized to the curve of the blank. The area under the fluorescence decay curve (AUC) was calculated as the following equation:

$$\text{AUC}=1+\sum_{i=1}^{i=120}fi/f0$$

Where *f*0 is the initial fluorescence reading at 0 min and *f*i is the fluorescence reading at time i. The net AUC was calculated by the expression: AUC_sample_—AUC_blank_. Regression equations between net AUC and Trolox concentration were calculated. ORAC-FL value of the tested compound expressed as Trolox equivalents.

#### 3.2.2 Inhibition of Aβ_1–42_ aggregation assay

Following the previous literature (Rosini et al., 2008; Wang et al., 2018), Aβ_1−42_ (Sigma-Aldrich) was dissolved in NH_4_OH (1% v/v) to get a stock solution, which was aliquoted into small samples and stored at −80°C.

#### 3.2.3 Metal-chelating study

**6b** and **6c** (50 µM) were incubated with CuSO_4_, FeSO_4_, FeCl_3_, or ZnCl_2_ (50 µM) in buffer (20 mM HEPES, 150 mM NaCl, pH 7.4) for 30 min, and the absorption spectras were recorded at room temperature. For the stoichiometry of the compound–Cu^2+^ complex, a fixed amount of **6b** and **6c** (50 µM) was mixed with growing amounts of copper ion (0–100 µM), and the difference UV−vis spectra were examined to investigate the ratio of ligand/metal in the complex.

#### 3.2.4 Statistical analysis

Data were presented as mean ± standard deviation (SD) (represented by error bars). All the experiments had three replicates (*n* = 3). *In vivo* anti-tumor Student’s *t*-test was used for comparing two groups, and significant differences were indicated by *p < 0.05, *p < 0.01, ***p < 0.001. The statistical analysis was performed with GraphPad Prism 8.0.1.

## 4 Conclusion

In this study, a series of novel melatonin–hydroxyquinoline hybrids were designed and synthesized, simultaneously targeting anti-oxidation and metal-chelating. Most of the compounds possess good blood-brain barrier permeability and showed significant oxygen radical absorbance capacity and Aβ_1–42_ aggregation inhibition. Among them, **6b** and **6c** have a good ability to alleviate oxidative stress (Figure 2C) induced by hydrogen peroxide and exhibit metal-chelating properties with the chelation ratio being 2:1. Furthermore, **6b** can significantly mitigate metal ion induced Aβ aggregation.

## Data Availability

The datasets presented in this study can be found in online repositories. The names of the repository/repositories and accession number(s) can be found in the article/Supplementary Material.
