# Supplementary material for: Discovery of novel melatonin–mydroxyquinoline hybrids as multitarget strategies for Alzheimer’s disease therapy
Source: Front Chem. 2024 Apr 16;12:1374930. doi: 10.3389/fchem.2024.1374930 (PMC11059093; doi:10.3389/fchem.2024.1374930)
Supplement: Supplementary file 1 [file DataSheet1.PDF]

Supplementary Material

Melatonin–Hydroxyquinoline Natural Products Hybrids as Multitarget Strategies for Alzheimer's Disease Therapy by Simultaneously Targeting Anti-oxidation and Metal Ion Chelation

Wei Wang[1], Tingting Pan[2], Rui Su[1], Mingbin Chen[1], Wandi Xiong[1], Congjun Xu[1*], Ling Huang[1*].

[1]Key Laboratory of Tropical Biological Resources of Ministry of Education, School of Pharmaceutical Sciences, Hainan University, Haikou, China

[2]School of Pharmaceutical Sciences, Sun Yat-sen University, Guangzhou 510006, China

*** Correspondence:**
Corresponding Author
congjunxu@hainanu.edu.cn (C. Xu); Linghuang@hainanu.edu.cn (L. Huang)

Contents:

Structural characterization of the compounds

Biological assays

^{1}H-NMR and ^{13}C-NMR of compound 3a

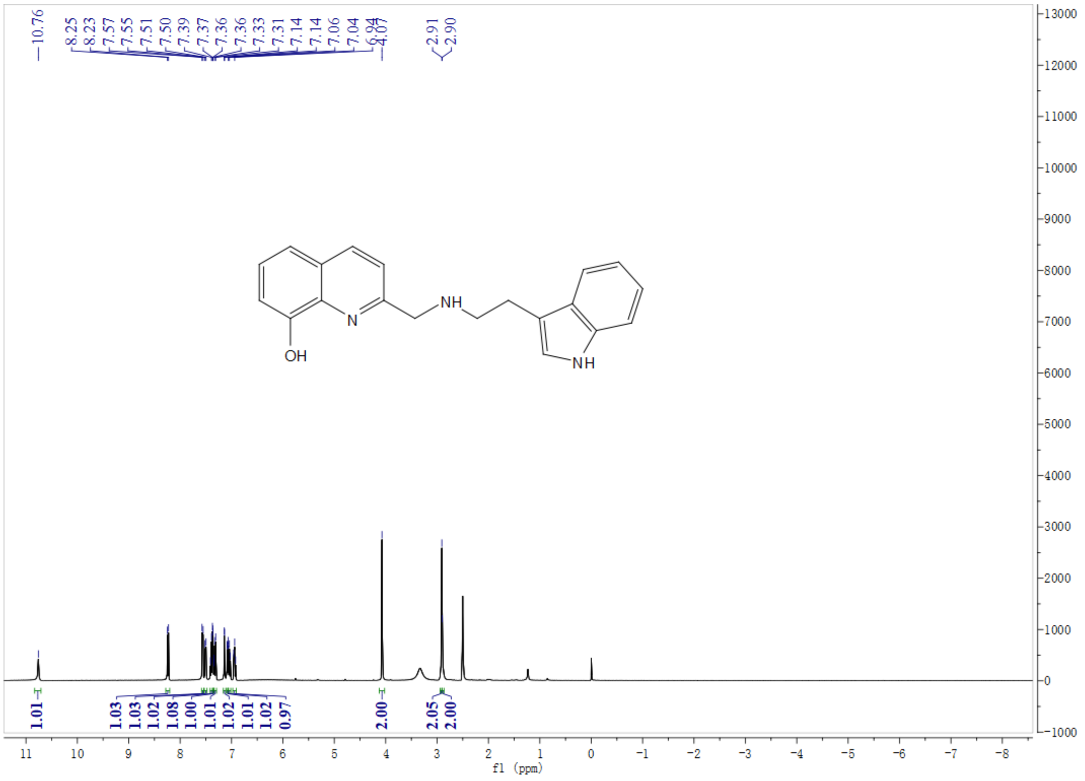

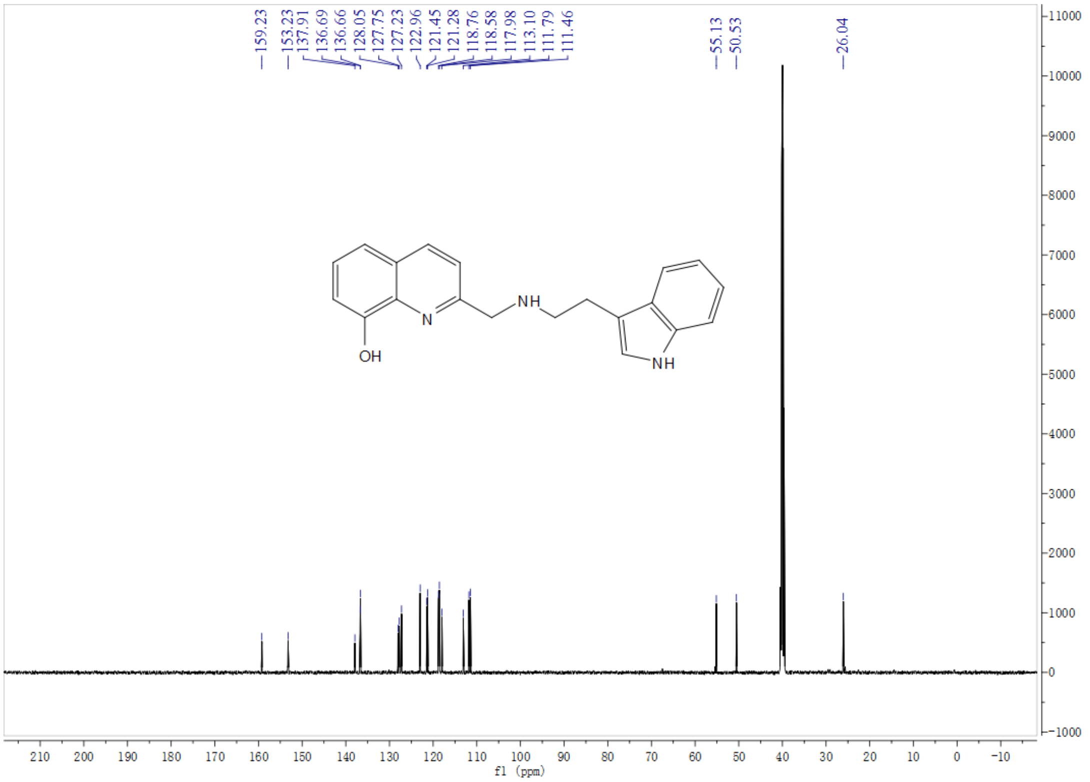

^{1}H-NMR and ^{13}C-NMR of compound 3b

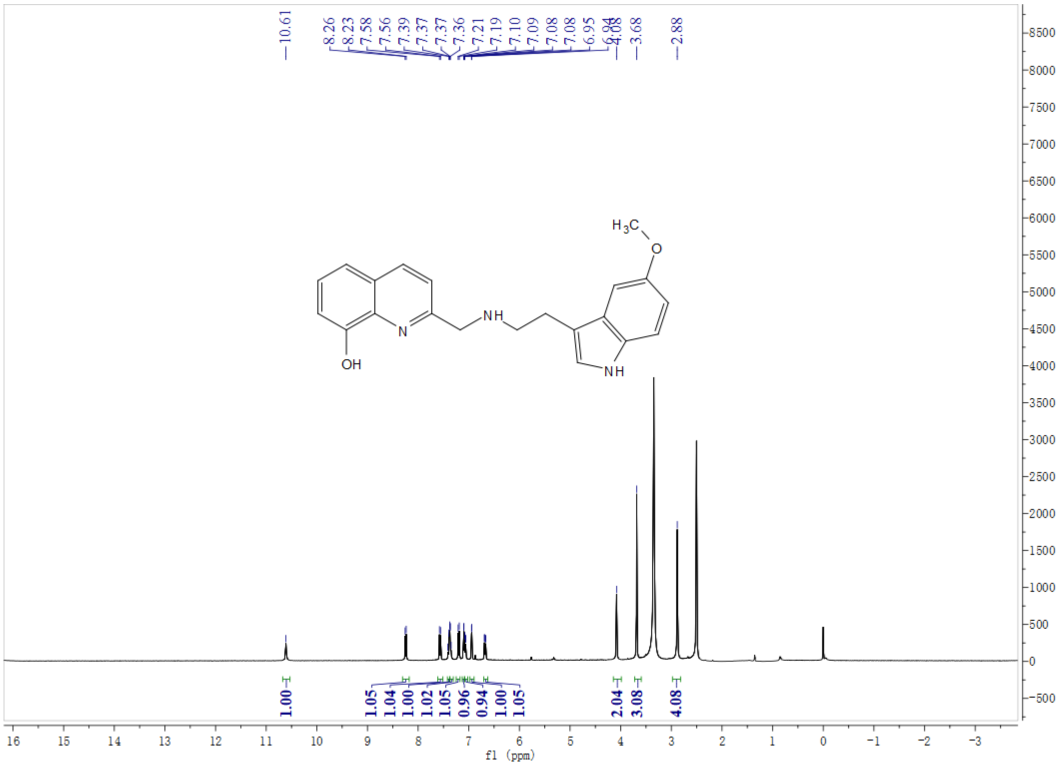

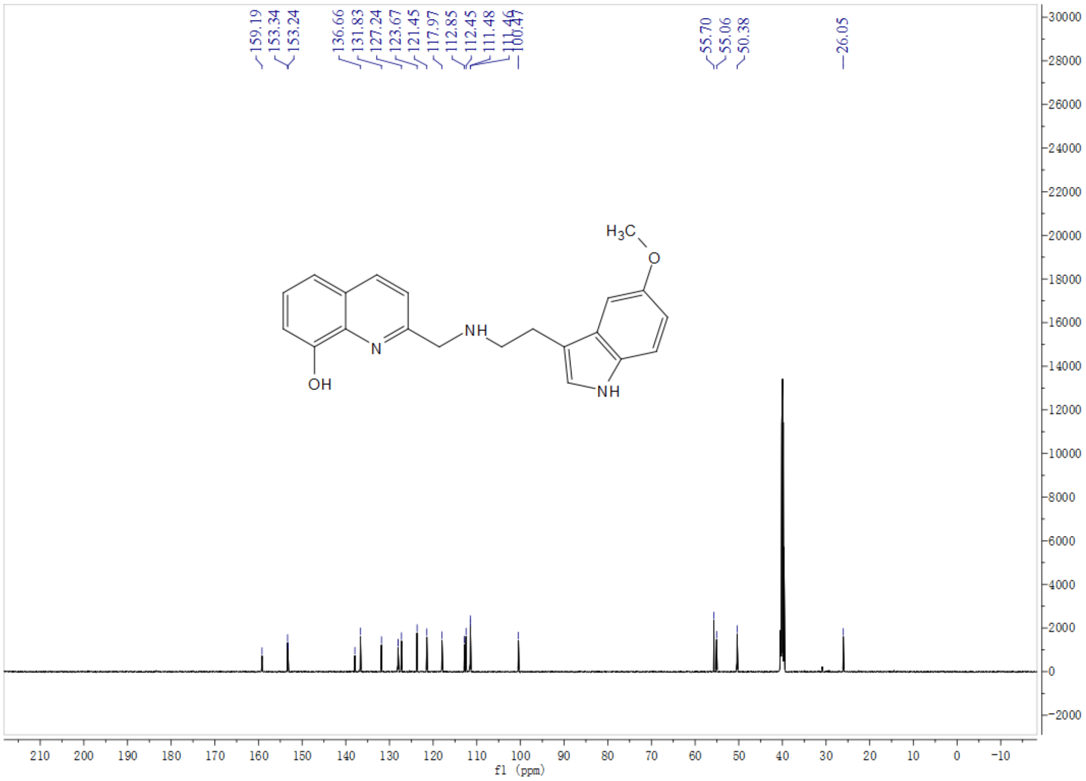

^{1}H-NMR and ^{13}C-NMR of compound 3c

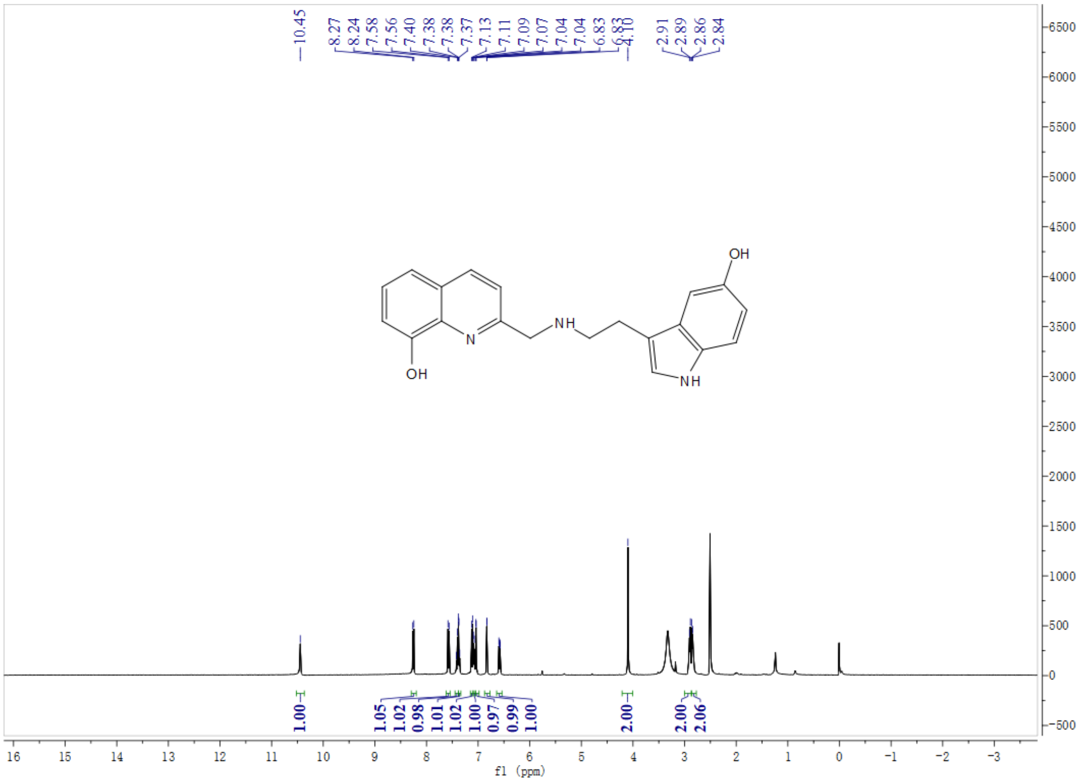

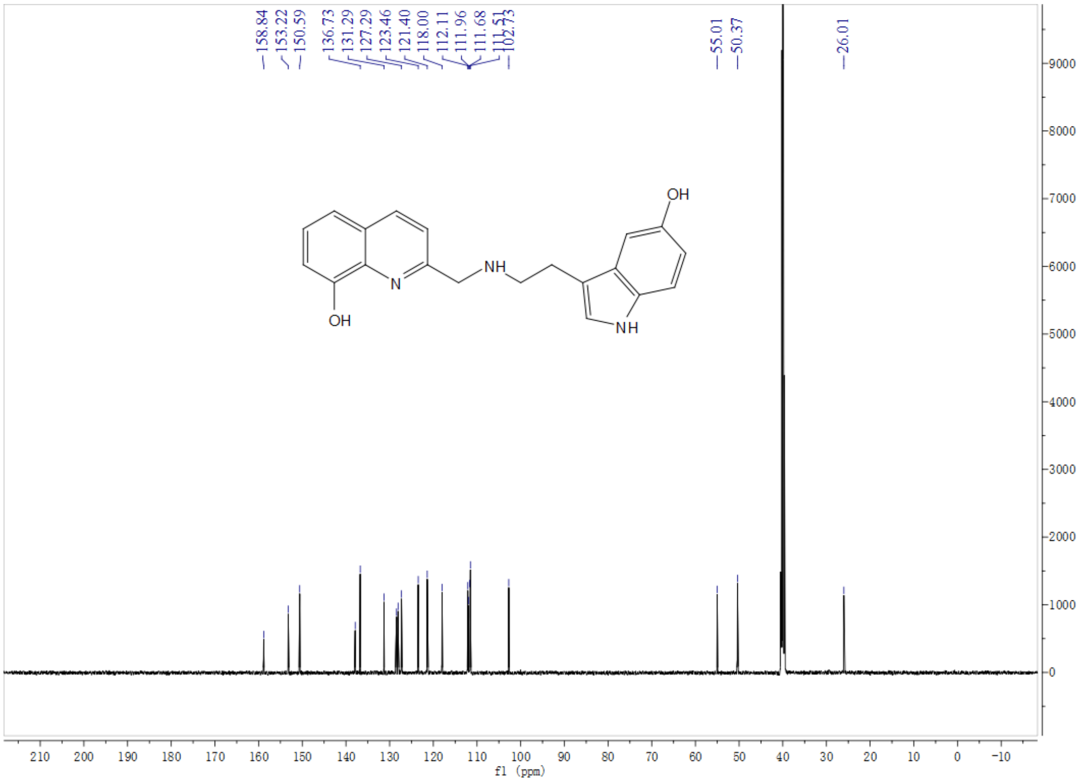

^{1}H-NMR and ^{13}C-NMR of compound 3d

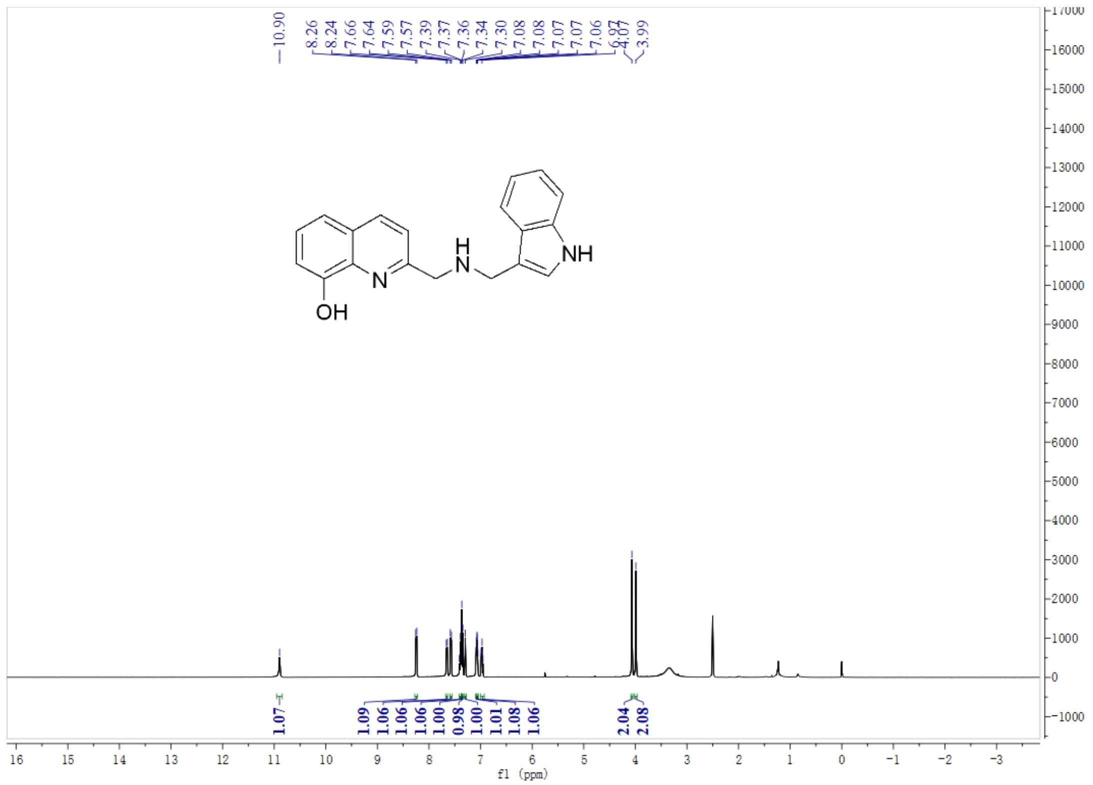

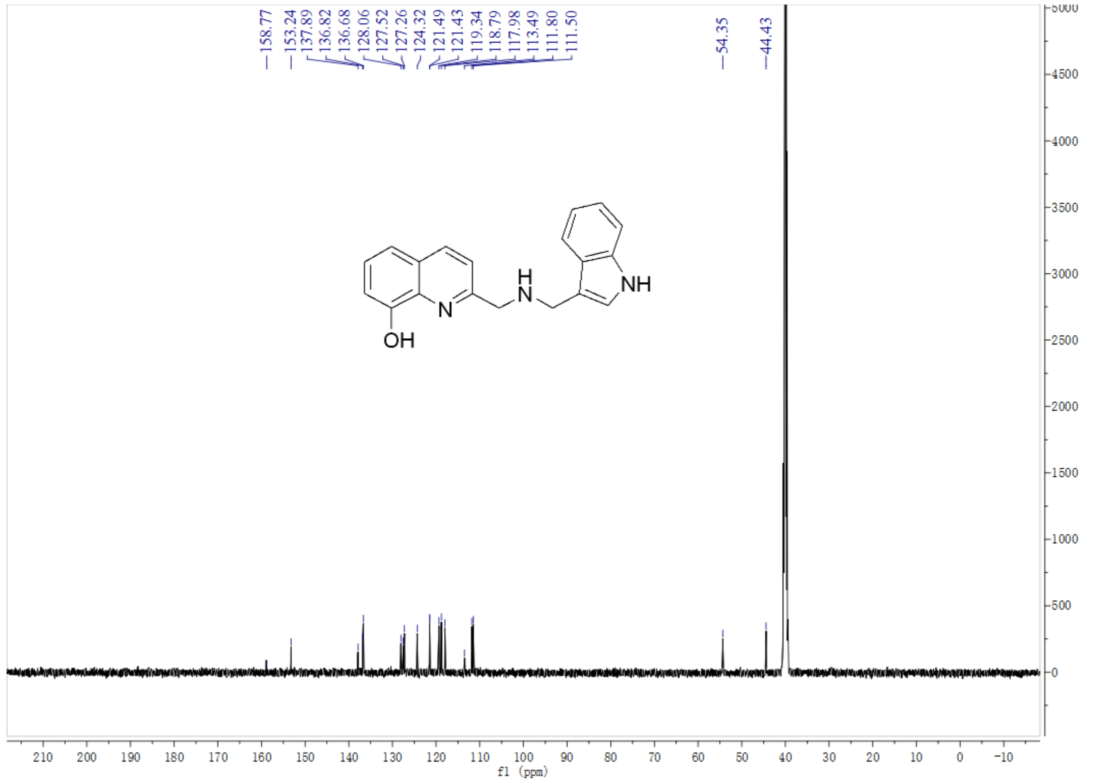

^{1}H-NMR and ^{13}C-NMR of compound 4

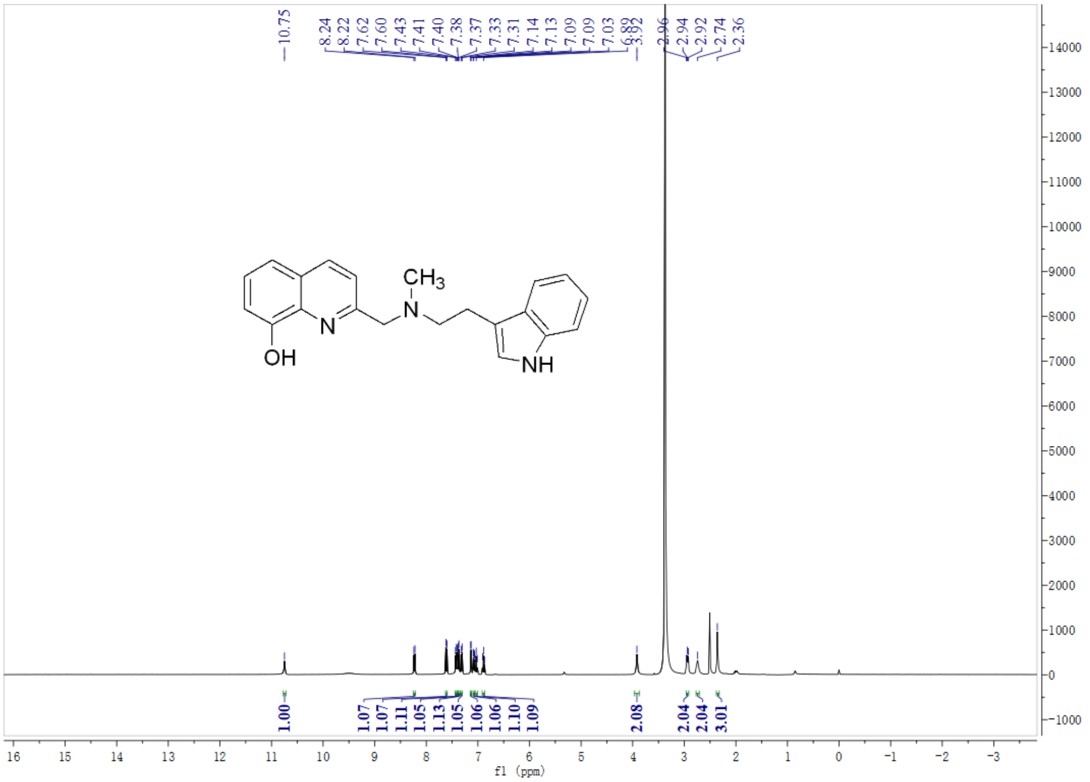

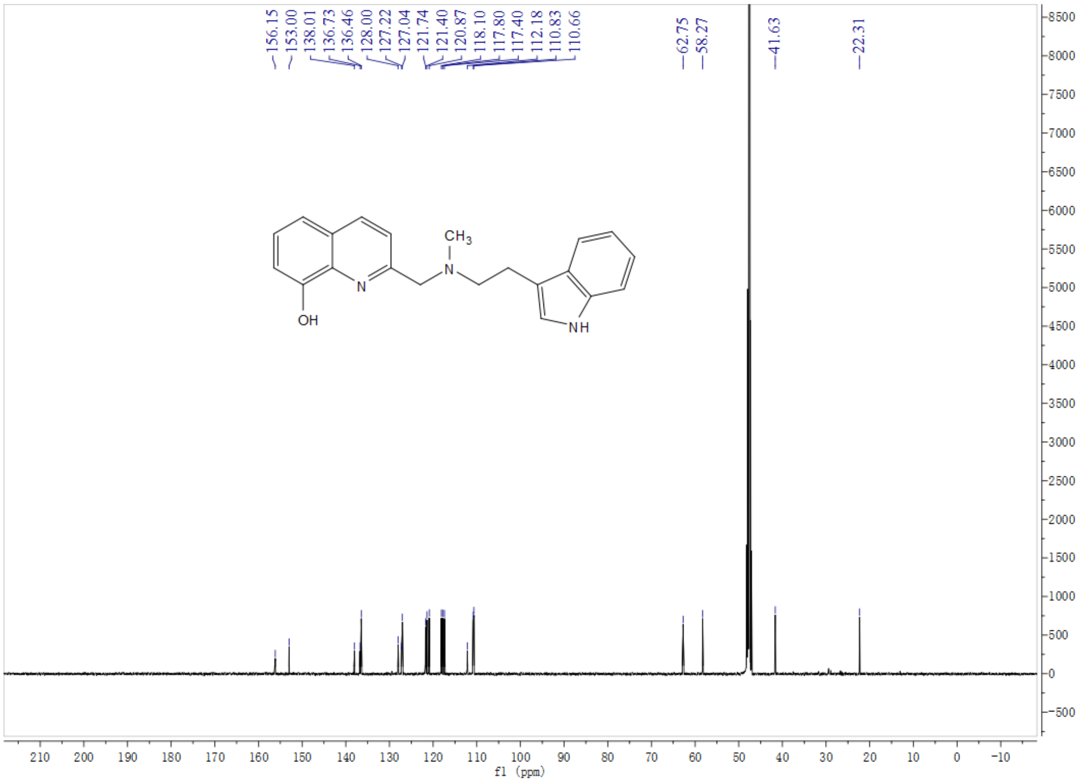

^{1}H-NMR and ^{13}C-NMR of compound 6a

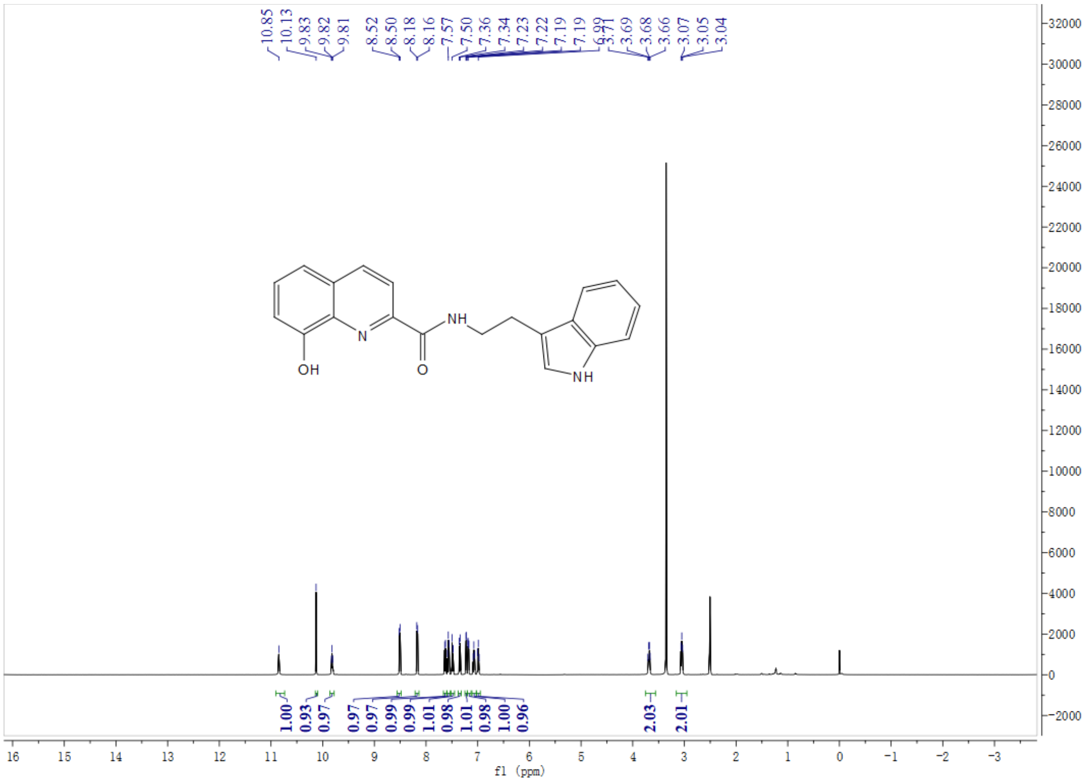

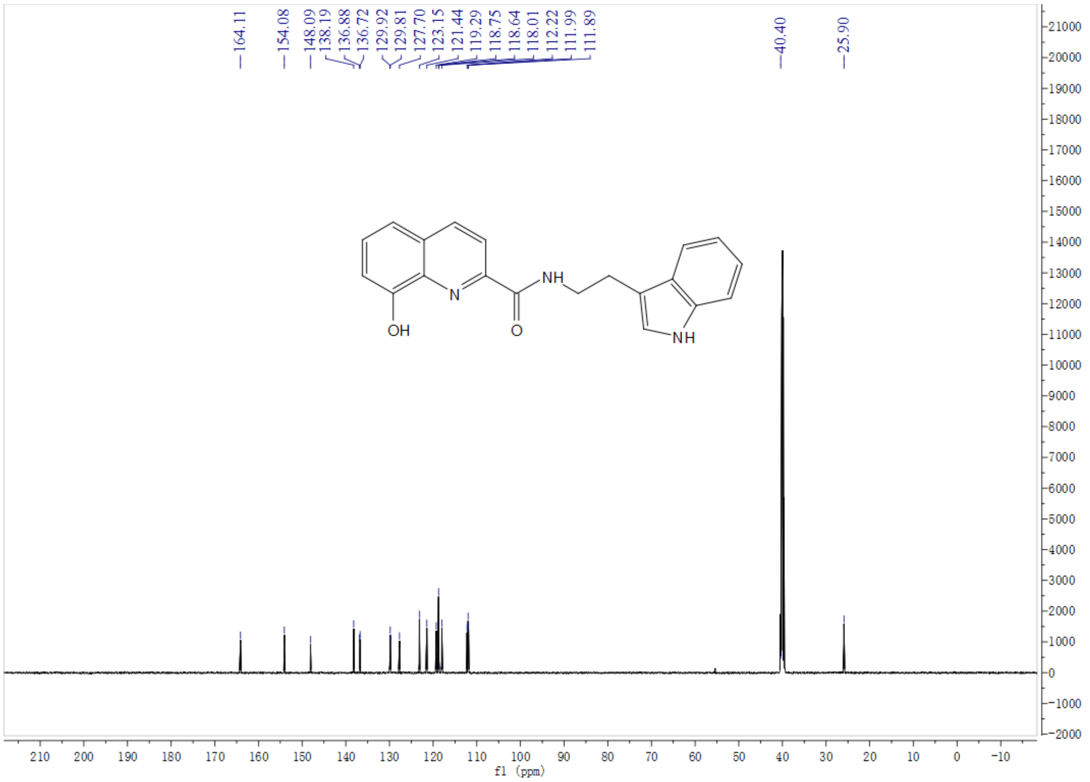

^{1}H-NMR and ^{13}C-NMR of compound 6b

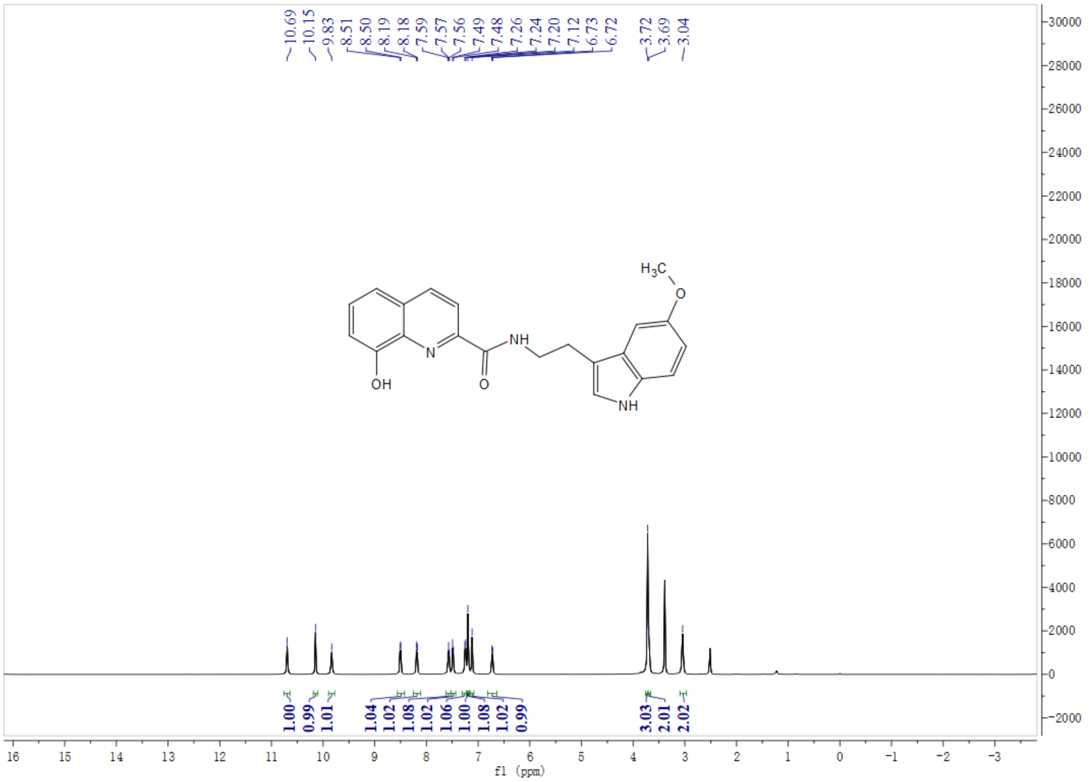

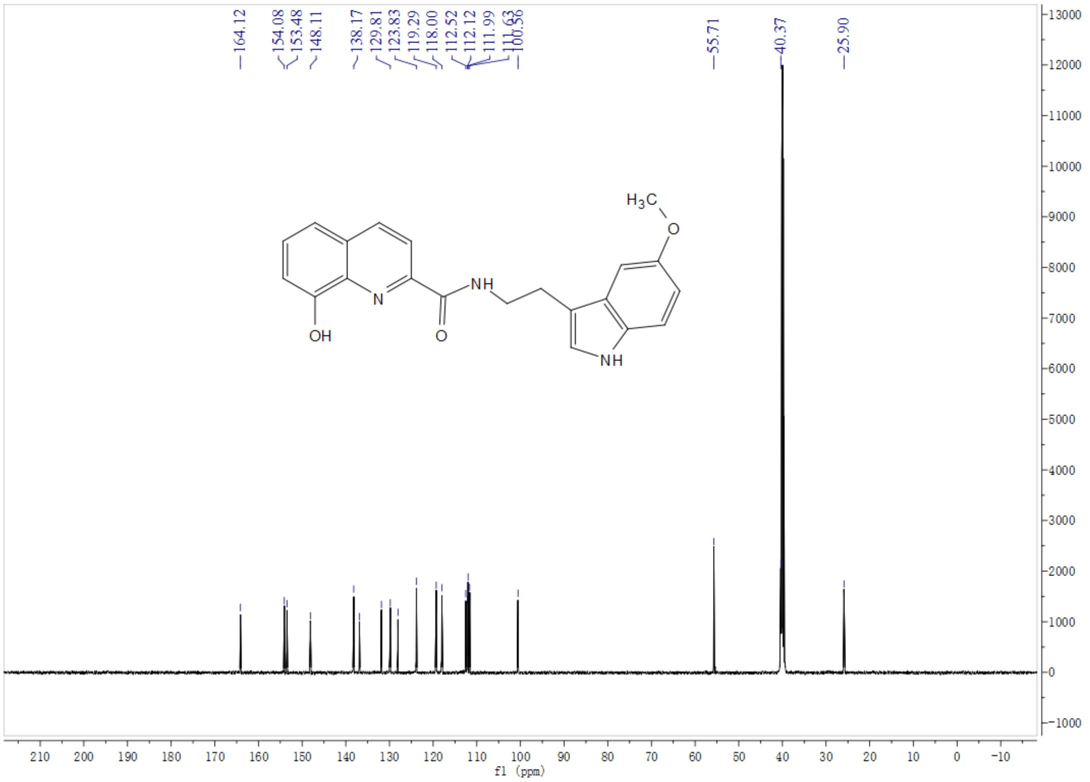

^{1}H-NMR and ^{13}C-NMR of compound 6c

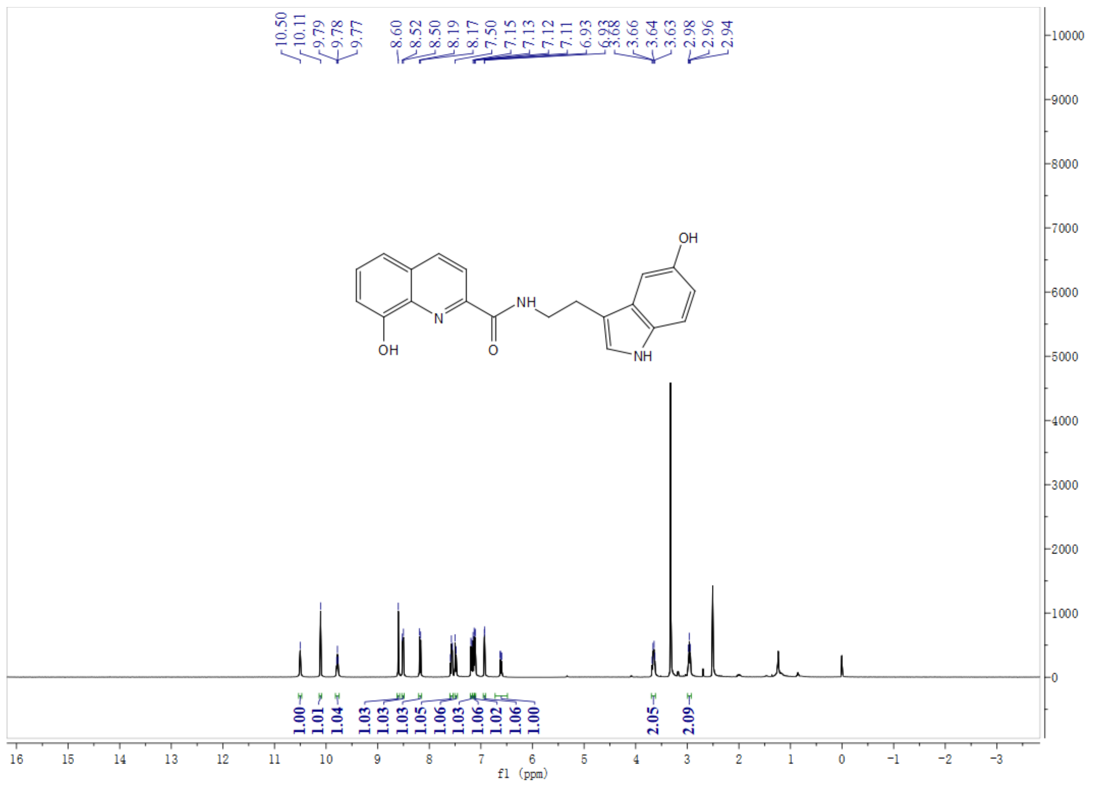

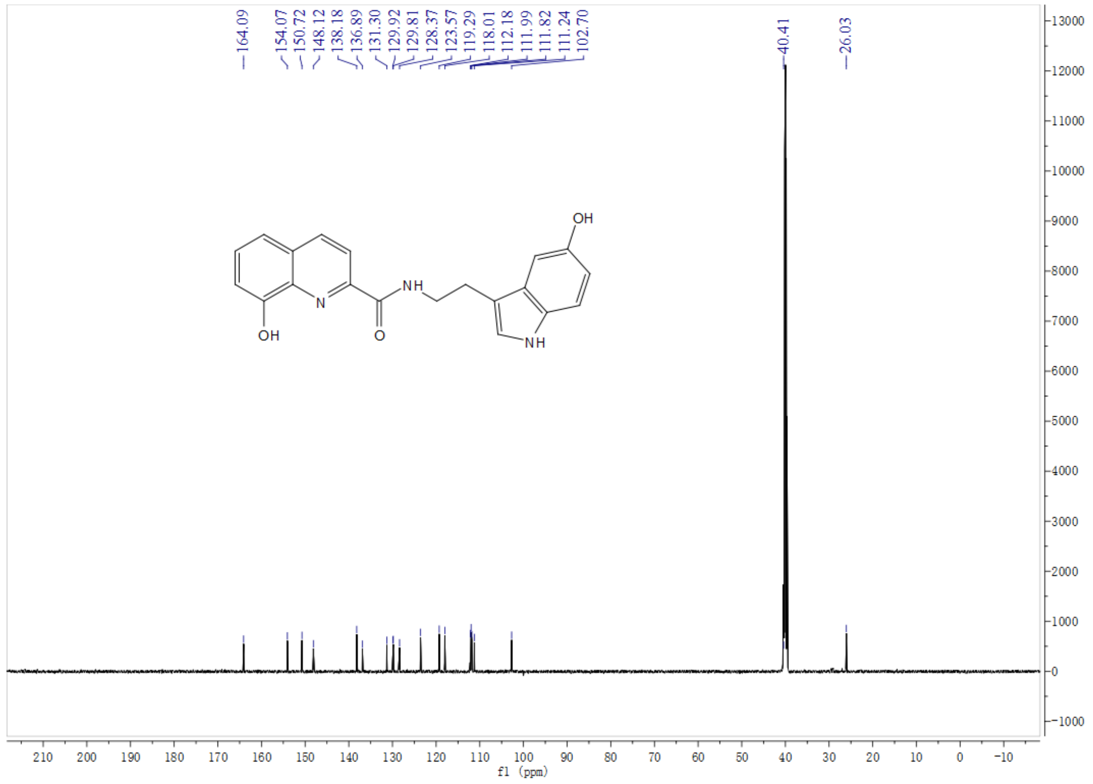

^{1}H-NMR and ^{13}C-NMR of compound 6d

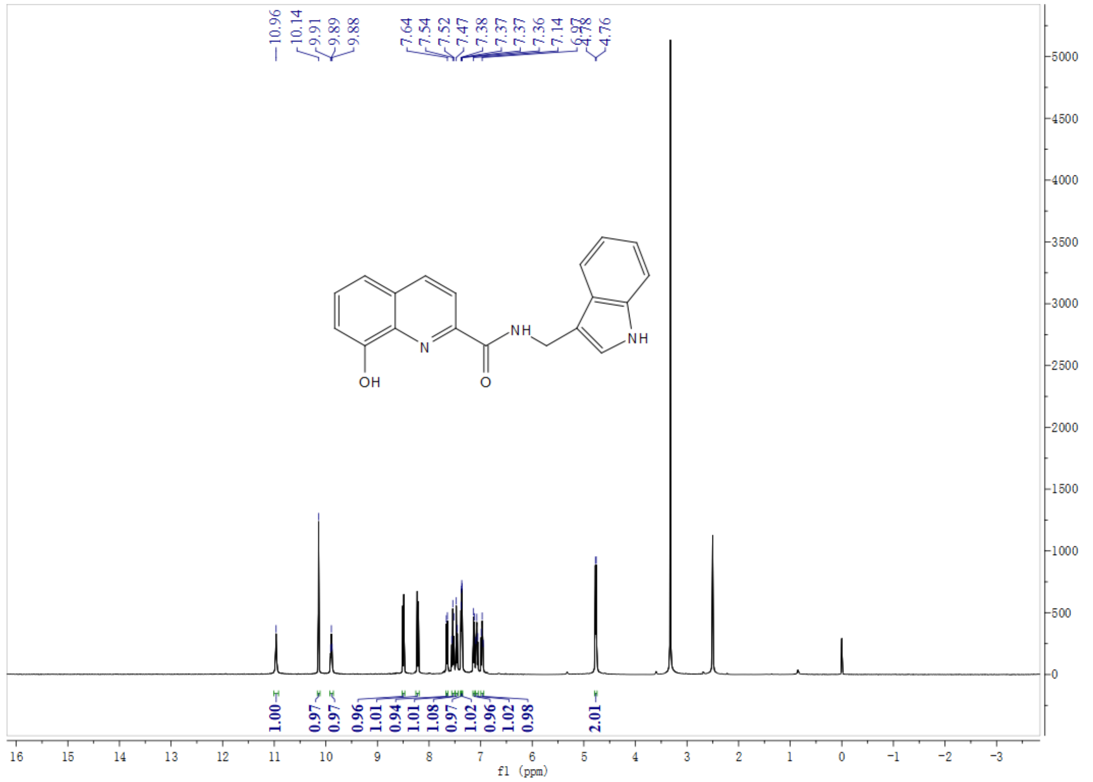

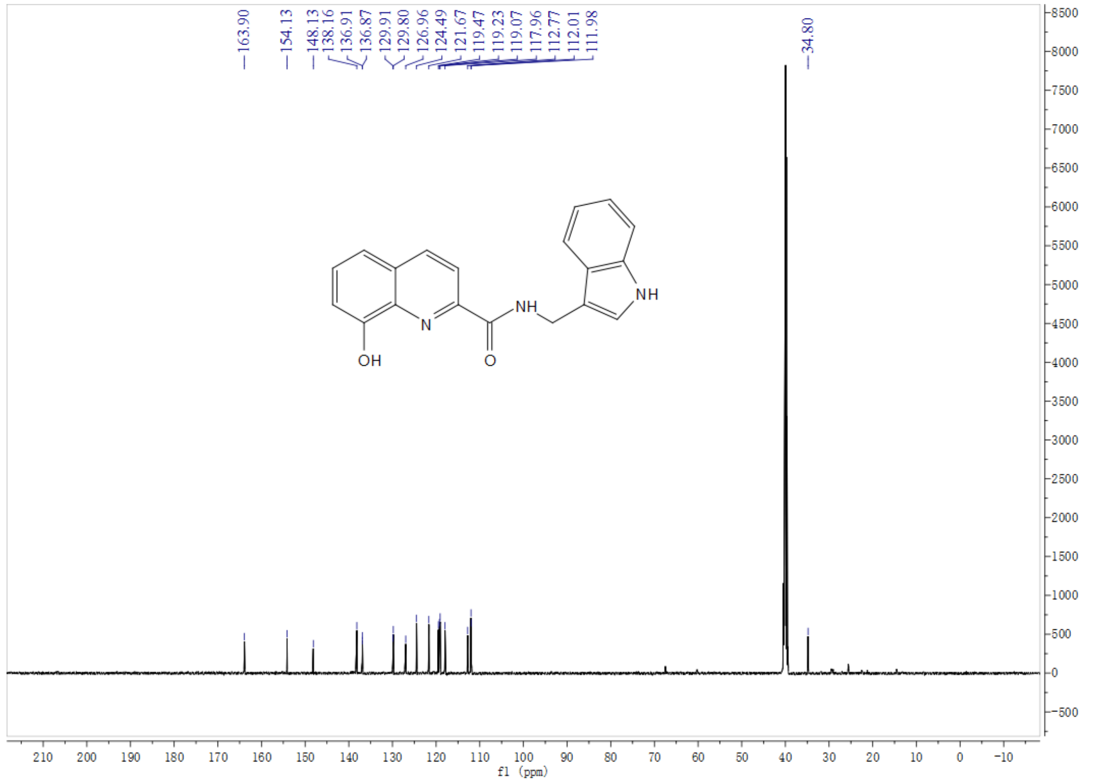

^{1}H-NMR and ^{13}C-NMR of compound 11a

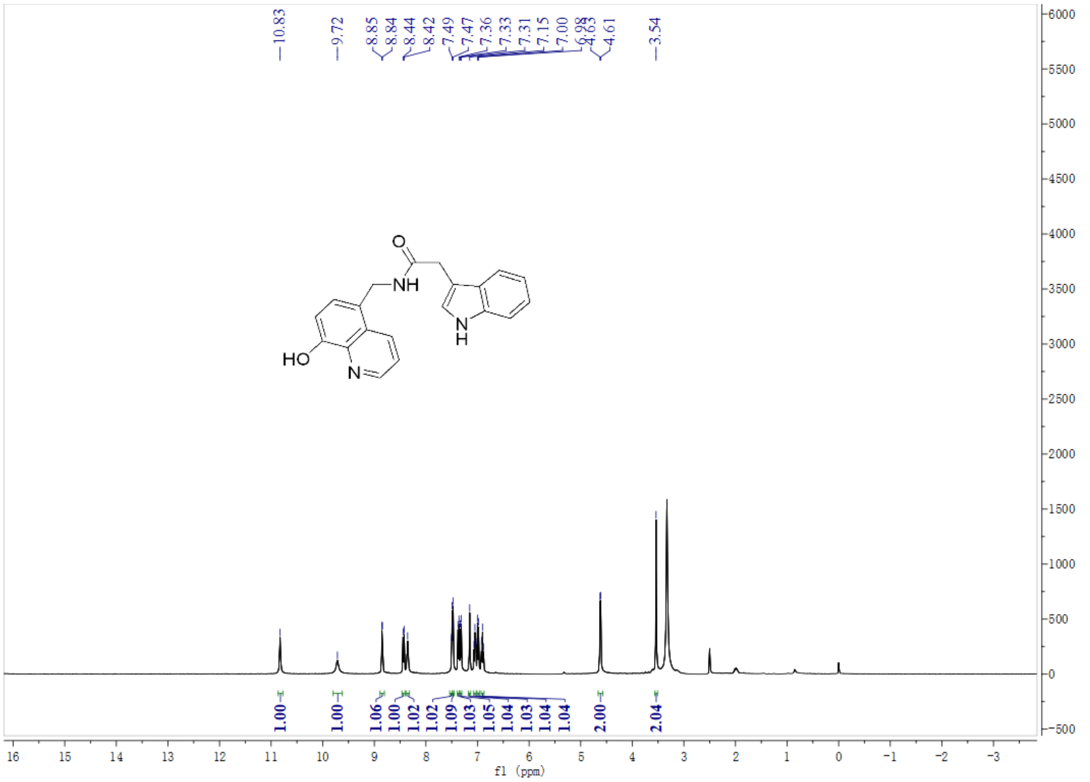

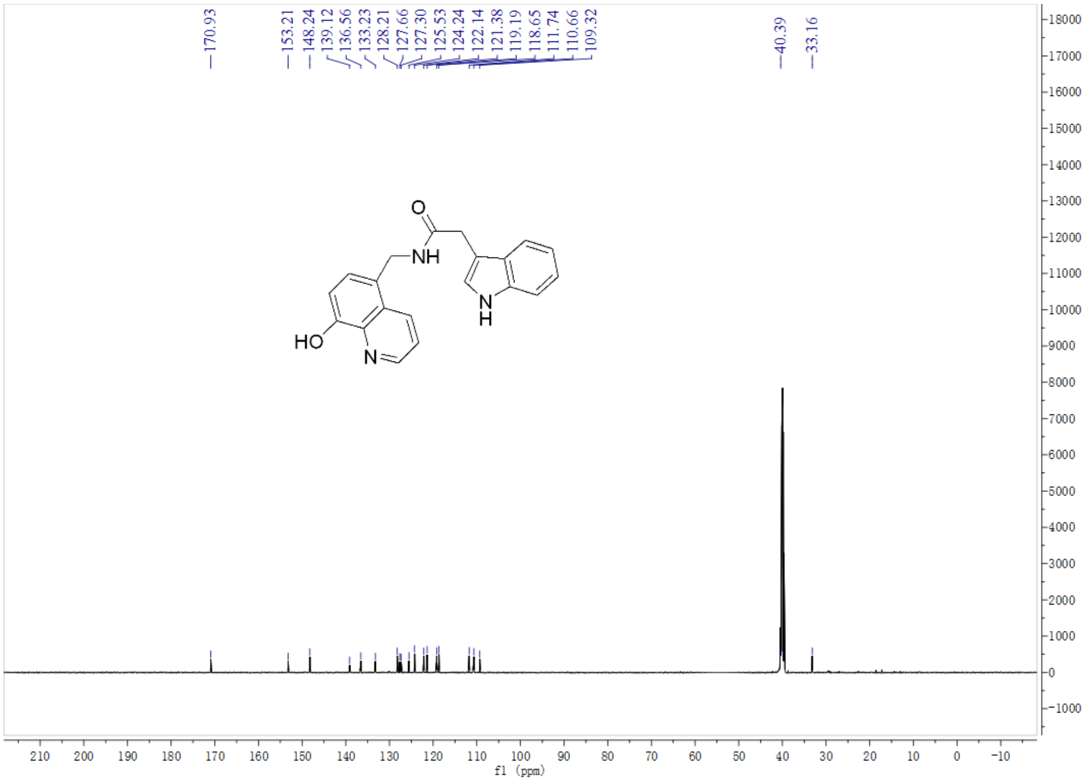

^{1}H-NMR and ^{13}C-NMR of compound 11b

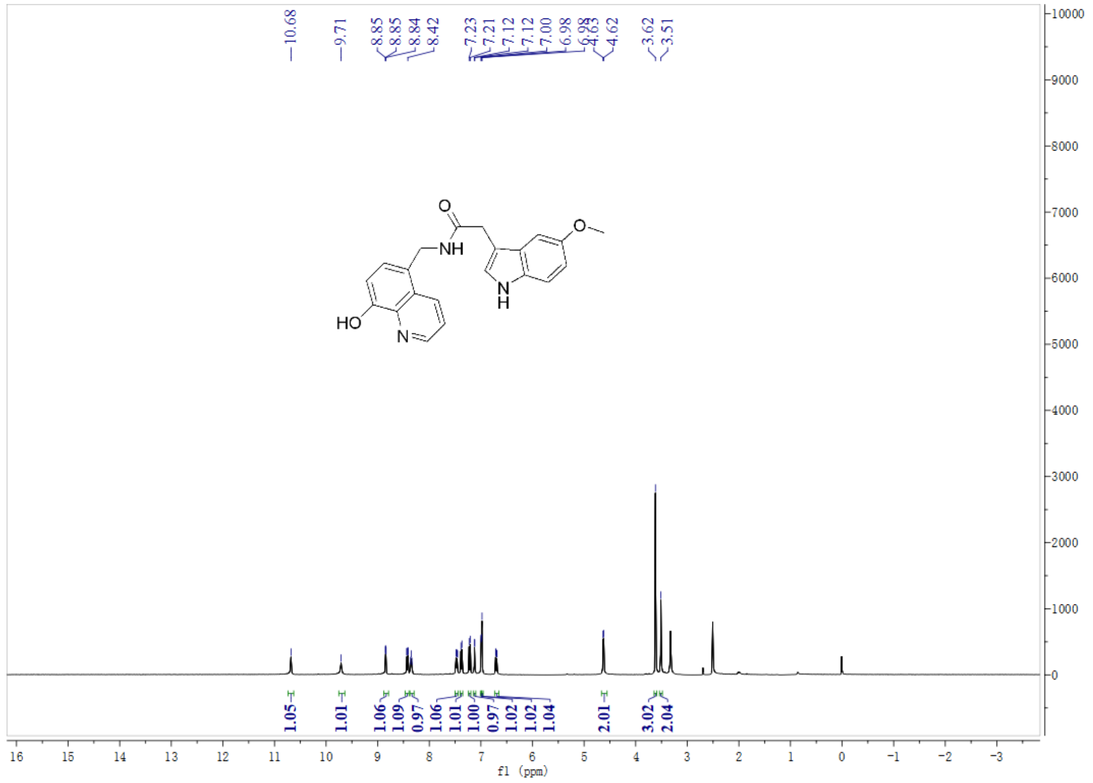

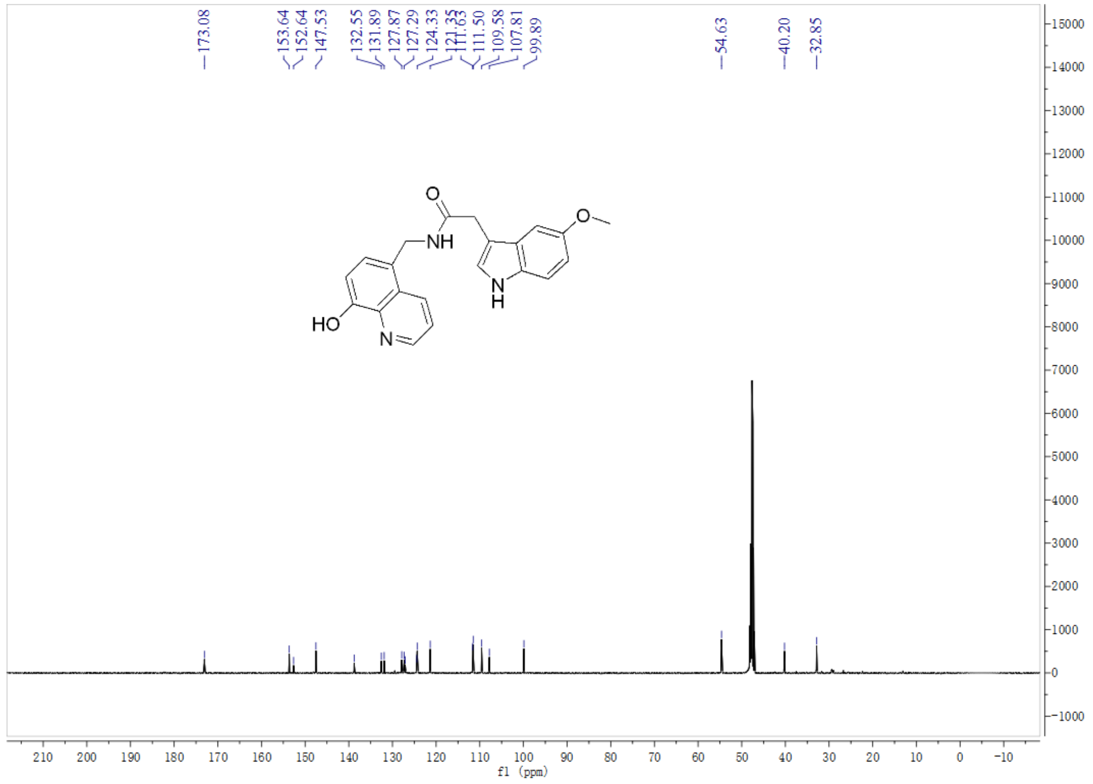

^{1}H-NMR and ^{13}C-NMR of compound 13a

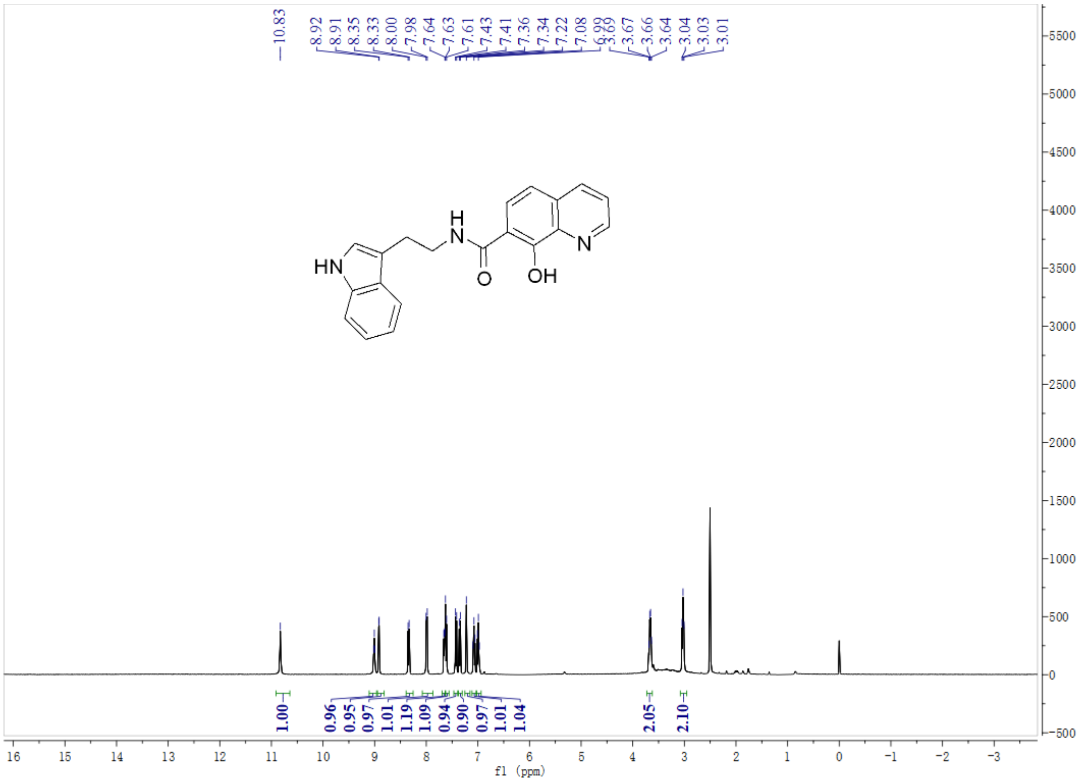

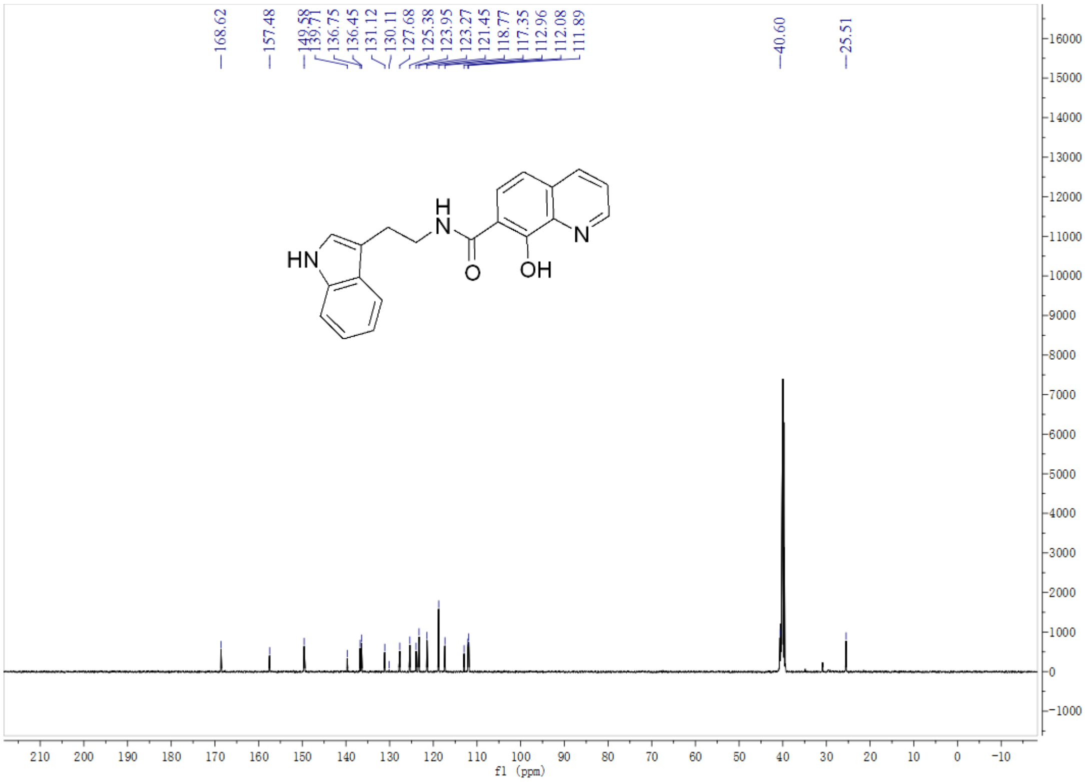

^{1}H-NMR and ^{13}C-NMR of compound 13b

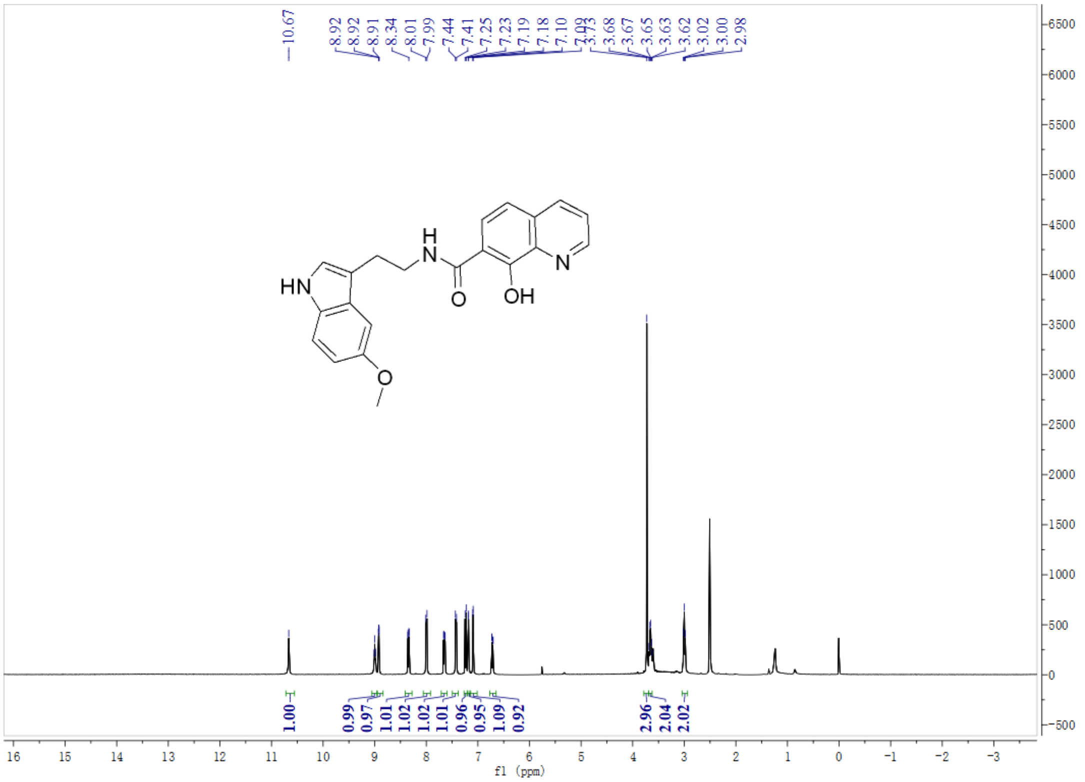

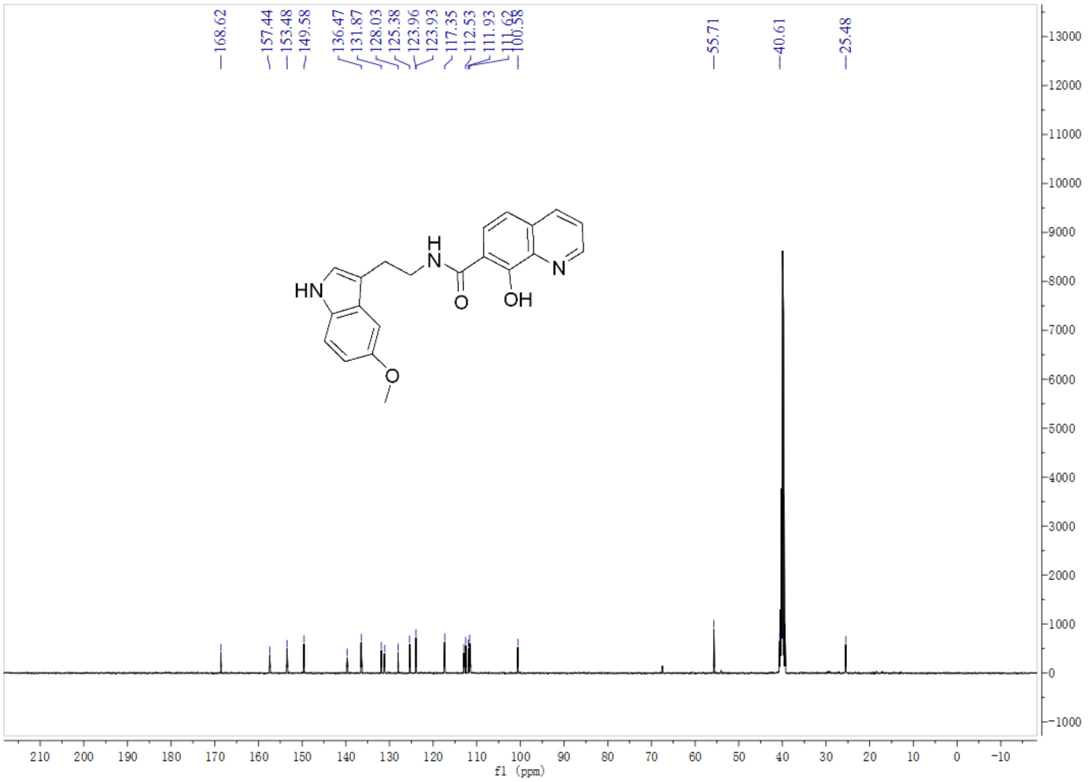

^{1}H-NMR and ^{13}C-NMR of compound 13c

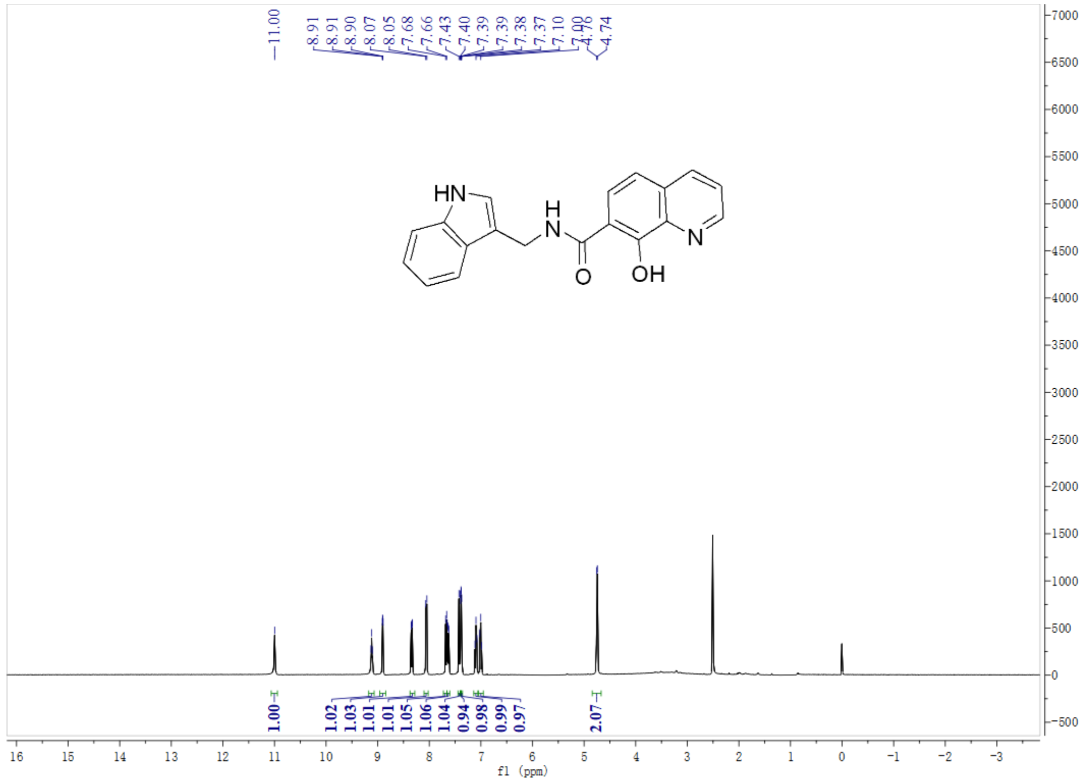

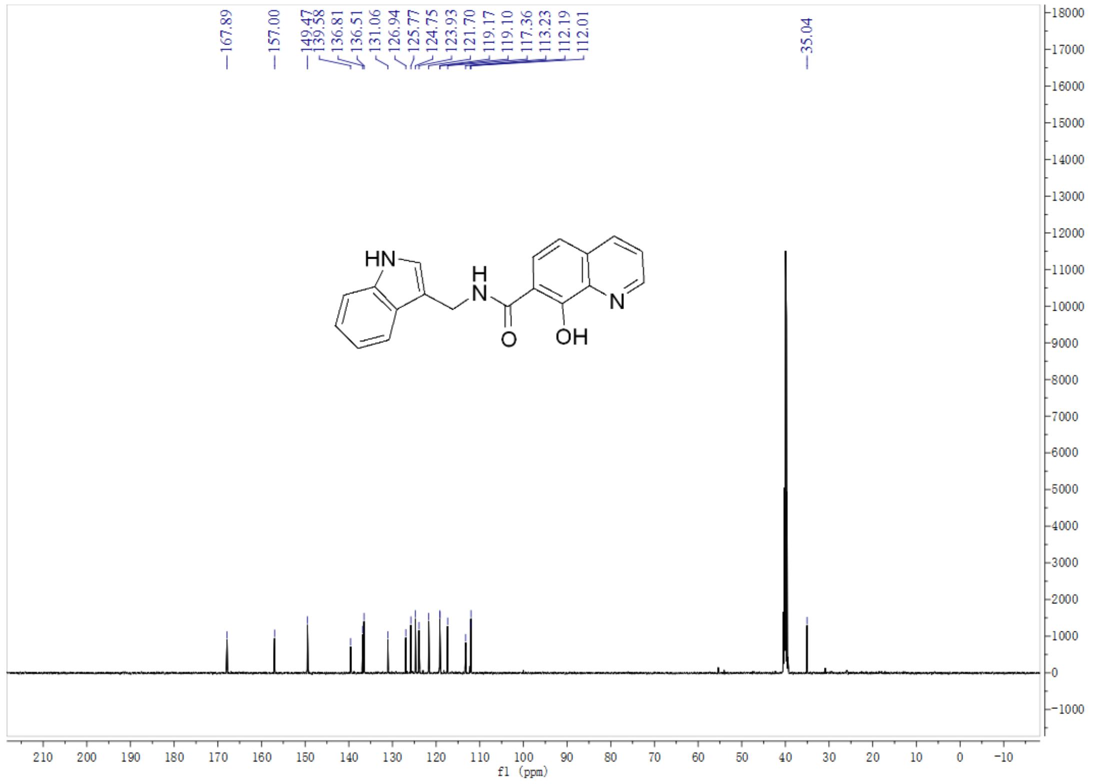

Table S1 Permeability ($Pe\times10^{-6}$ cm s^{-1}) in the PAMPA-BBB assay for 13 commercial drugs, used in the Experiment Validation

**Drug**	**Bibl** [a]	**PBS : EtOH (70 : 30)** [b] **(×10^{-6} cm s^{-1})**
testosterone	17	22.3 ± 1.4
verapamil	16	21.2 ± 1.9
desipramine	12	16.4 ± 1.2
progesterone	9.3	17.7 ± 1.2
promazine	8.8	14.3 ± 0.5
chlorpromazine	6.5	6.0 ± 0.3
clonidine	5.3	5.1 ± 0.3
piroxicam	2.5	0.24 ± 0.01
hydrocortisone	1.9	0.65 ± 0.01
lomefloxacin	1.1	0.37 ± 0.02
atnolol	0.8	0.78 ± 0.02
ofloxacin	0.8	0.37 ± 0.02
theophylline	0.1	0.26 ± 0.01

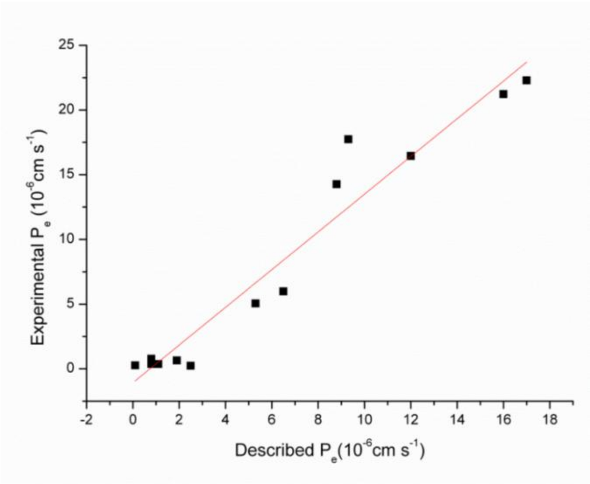

Figure S1 Lineal correlation between experimental and reported drugs.

Table S2 Ranges of Permeability of PAMPA-BBB Assays (P_e, 10^{-6} cm s^{-1}).

Compounds of high BBB permeation (CNS+)	$P_e>4.7$
Compounds of uncertain BBB permeation (CNS+/-)	$4.7>P_e>1.8$
Compounds of low BBB permeation (CNS-)	$P_e<1.8$
